# Archaeal tubulin-like proteins CetZ1 and CetZ2 have opposing effects on cell morphology during the growth cycle of *Haloferax volcanii*

**DOI:** 10.1128/mbio.00854-26

**Published:** 2026-08-07

**Authors:** Hannah J. Brown, Iain G. Duggin

**Affiliations:** 1 The Australian Institute for Microbiology and Infection, University of Technology Sydney, Ultimo, NSW, Australia; NYU Langone Health, New York, New York, USA

**Keywords:** archaea, cytoskeleton, cell shape, tubulin, CetZ

## Abstract

CetZs are archaeal tubulin superfamily cytoskeletal proteins implicated in the control of cell shape and motility. In the pleomorphic archaeon *Haloferax volcanii*, CetZ1 is required for the transformation of a discoid, or plate-like, cell morphology to the rod shape in early log-phase cultures, and for the development of swimming motility. In this study, we found that the paralog CetZ2 is not required for rod development or reversion to plates in log phase but was strongly upregulated later in stationary phase, where it participates in the maintenance of plate cell shape. Overproduction of CetZ2 or a functional fluorescently tagged CetZ2 promoted maintenance of plate cell shape specifically in the stationary phase. The tagged CetZ2 localized in patchy subcellular assemblies at the cell edges that were most dynamic in mid-stationary phase, where they showed directional movement around the cell edge and other complex behaviors. The stationary phase dynamics of CetZ1 and CetZ2 localization patterns were dependent on their individual GTPase activities and on the presence of one another, despite the much higher cellular concentration of CetZ1 over CetZ2 (ratios of over 40:1 in log and 5:1 in stationary phase). Together, the results suggest that CetZ2 counteracts the CetZ1-based rod development pathway to maintain plate shape in the stationary phase and imply that additional stationary-phase factors are required. CetZ1 and CetZ2 are co-conserved in many haloarchaea, suggesting that their coordinated functions at different stages of the growth cycle may be widely utilized to control cell shapeshifting.

**IMPORTANCE:**

This study describes the first defined function of a CetZ2 protein, which represents a distinct haloarchaeal protein family within the near-universal tubulin superfamily of cytoskeletal proteins. Using the model archaeal organism *Haloferax volcanii*, we showed that CetZ2 is upregulated as cultures enter the stationary phase, where it dynamically localizes in patches at the cell edges and maintains the plate- or disk-like cell morphology, thus counteracting the CetZ1-based rod cell development. This CetZ1-CetZ2 interplay might represent a useful paradigm for understanding the evolution of antagonistic cytoskeletal functions, such as those that led to a reliance on active and inactive subunits during the early evolution of microtubules in eukaryotes. Our findings also suggest that diversification and specialization of tubulin homologs have occurred multiple times in archaeal evolution.

## INTRODUCTION

CetZs are archaea-specific tubulin superfamily proteins that are structural homologs of eukaryotic tubulin, which forms microtubules, and FtsZ, the key cell division protein present in both bacteria and archaea (1, 2). One characteristic of these tubulin superfamily proteins is that they polymerize in a GTP-dependent manner, forming filaments or large polymers (3–9), which can assemble into larger cytoskeletal structures that carry out their biological functions. Tubulin superfamily proteins, including CetZ (1), typically show dynamic remodeling in cells due to their capacity to polymerize and depolymerize in a regulated GTPase-dependent manner, which is central to their cytoskeletal functions. This commonly manifests as polymer treadmilling, where the protein subunits assemble directionally and simultaneously disassemble from the other end. FtsZ and tubulin have a wide diversity of functions which are well studied, including in cell division and chromosome segregation (10, 11), intracellular trafficking and cargo transport (12, 13), and cell motility or migration (14, 15).

CetZs are conserved across many diverse archaea. In Halobacteria (Haloarchaea), it is typical that one species would have multiple paralogs of CetZ. The most highly conserved of these are CetZ1 and CetZ2 (1, 16, 17), which form distinct phylogenetic clusters, suggesting that CetZ1 and CetZ2 could have different functions. While both CetZ1 and CetZ2 have been implicated in the control of shape and motility in *Haloferax volcanii*, only CetZ1 currently has a clearly defined biological function. During the early stages of standard batch culture, *H. volcanii* transitions from plate-shaped cells to rods, and then in later growth and stationary phases, they revert to plates (18). CetZ1 is necessary for the early-stage rod development (1, 18) but is also required for rod cell development and elongation in other conditions, such as in motility agar (1, 19, 20) or when cultures are depleted of trace elements (18). CetZ1 is thought to contribute to motility by generating the rod shape that enables directional and streamlined swimming, as well as by promoting the positioning and assembly of archaella and chemosensory arrays near the cell poles (21).

CetZ1 is a cytoplasmic protein but localizes in patches that appear to line the cell envelope, and it is often concentrated at mid-cell. It also localizes at cell poles under the influence of MinD subcellular positioning systems (22) and is hypothesized to participate in the formation of a cap-like structure in motile rods (1, 20–23). CetZ1 localization during growth and rod development is also remarkably dynamic, involving apparent movement and continual reassembly in cells. This has been linked to its polymerization and depolymerization cycle (1, 23), since a point mutation in the GTPase active site (CetZ1.E218A, based on the equivalent mutations in FtsZ and tubulin), causes it to form the expected hyper-stable structures *in vivo*, thus disrupting CetZ1 assembly dynamics that are important to its function (1). Expression of CetZ1.E218A also prevents rod development, even in the presence of the endogenous CetZ1, and produces cells that show local regions of high envelope curvature, or “jagged” cells, where CetZ1.E218A localizes to the regions of high membrane curvature (1, 23).

While CetZ1 has roles in cell shape control and motility linked to its dynamic assembly patterns, no clear role of other CetZ paralogs of *H. volcanii* has been identified. Although CetZ2 has been generally implicated in the control of cell shape and motility (1, 21), deletion of *cetZ2* results in no cell division, shape, or motility phenotypes during exponential phase or on soft agar, respectively (1, 21). However, overexpression of CetZ2 induces a slight hypermotility phenotype and promotes assembly of archaella (21). In addition, expression of the GTPase mutant CetZ2.E212A prevents rod development and perturbs motility (1, 21). CetZ2.E212A might therefore interfere with CetZ1 function (1), whether through direct interaction with CetZ1 or indirectly through an unknown mechanism. Together with the knowledge that CetZ2 is only present in Halobacteria that also have CetZ1 (17), the current evidence points toward a potential functional interplay between CetZ2 and CetZ1. One possible explanation for the lack of observable effects on cell shape and motility upon deletion of *cetZ2* is that the CetZ2 protein may be inactive or insufficiently produced under the previously tested conditions (i.e., early/mid-log growth and motility assays). Here, we show that CetZ2 is strongly upregulated in the stationary phase, where it maintains plate shape via a direct or indirect counteraction of CetZ1-dependent rod development.

## RESULTS

### CetZ2 is strongly upregulated in stationary phase in *H. volcanii*

**Fig 1:**
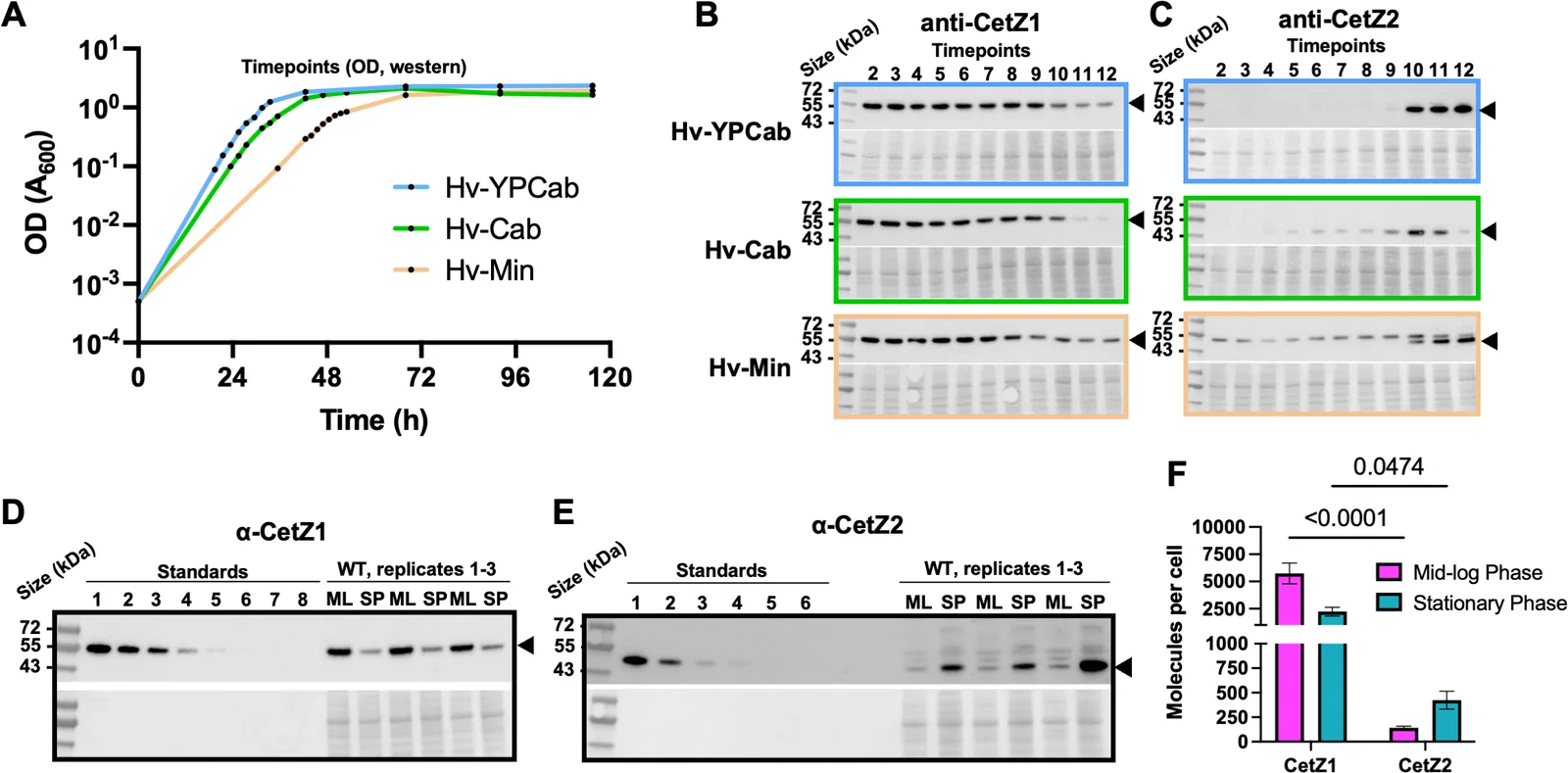
Quantification of CetZ1 and CetZ2 in *H. volcanii* throughout growth. (**A**) Wild-type cells (H26 + pTA962) were grown in Hv-YPCab, Hv-Cab, or Hv-Min from a theoretical starting OD_600_ ~0.0005. Data points indicate the time and OD_600_ measurements at which whole-cell lysate samples were taken for western blotting. (**B**) Western blot analysis of wild-type cells grown in panel **A** using antibodies against CetZ1 and (**C**) CetZ2. Samples from timepoints 2–12 of each medium were used for analysis. Ponceau S staining (lower section of each panel) was used as a sample loading control. Solid arrowheads indicate CetZ1/2 bands. Note that in Hv-MinTE medium (Hv-Min), a cross-reactive band appears in all lanes slightly above the CetZ2 band. (**D**) Purified CetZ1 was used to make eight standards of known concentrations. Standard 1 is 50 ng of pure CetZ1, and standards 2–8 are 1:2 serial dilutions of standard 1. Standards were compared to whole-cell lysate samples of wild-type cells grown in Hv-YPCab medium to estimate the number of CetZ1 molecules per cell in mid-log (L) and stationary (S) phases, 24 and 96 h after culture inoculation, respectively, using western blot detection of CetZ1. (**E**) As in panel D, except for CetZ2. Standard 1 is 1.25 ng of pure CetZ2, and standards 2–6 are 1:2 serial dilutions of standard 1. In panels D and E, six culture replicates were carried out, and the same samples were used for analysis of CetZ1 and CetZ2 expression levels. Solid arrowheads indicate CetZ1/2 bands. (**F**) From the results of panels D and E, the number of CetZ1/CetZ2 molecules per cell in mid-log and stationary phases was calculated as described in the methods. Mean and standard error are shown. One-way analysis of variance was used to assess significance.

CetZ2 function has previously been investigated during exponential phase growth and motility conditions. However, proteomic data (24) suggested that it may be upregulated in stationary phase in *H. volcanii* and *Natrialba magadii*. Here, we used western blotting to detect CetZ2 levels in Hv-YPCab (rich medium), Hv-Cab (casamino acids medium), and Hv-MinTE (minimal medium) between early log phase and late stationary phase of *H. volcanii* (Fig. 1A). CetZ1 was produced throughout the lag and log phases, though decreased in stationary phase (Fig. 1B). In contrast, there was little production of CetZ2 until stationary phase, where protein levels strongly increased (Fig. 1C). The downregulation of CetZ1 in stationary phase was not dependent on the presence of CetZ2 (Fig. S1A), and the upregulation of CetZ2 was not dependent on the presence of CetZ1 (Fig. S1B). The ratios between CetZ1 and CetZ2 and the number of molecules per cell were also measured using CetZ1/2 pure protein standards (Fig. 1D and E, respectively). Wildtype cells in mid-log growth were measured to have, on average, 5,700 molecules of CetZ1 and 140 molecules of CetZ2, giving a ratio of ~41:1 (CetZ1:CetZ2, Fig. 1F). In stationary phase, the amounts averaged 2,200 molecules of CetZ1 and 430 molecules of CetZ2 per cell, a ~5:1 ratio (Fig. 1F).

### CetZ2 contributes to maintenance of plate shape in stationary phase

CetZ2 has previously been implicated in cell shape through the expression of a GTPase active-site mutant (CetZ2.E212A) (1). This type of mutation (in the T7-loop) is expected to inhibit GTPase activity in tubulin-superfamily proteins, producing inactive hyper-stable polymers. The previous work showed that production of CetZ2.E212A resulted in a dominant inhibitory effect, preventing rod formation and causing distorted cell shapes, similar to the effects of the equivalent mutant of CetZ1 (E218A). However, this did not reveal the normal role of CetZ2 (1). Based on the above results, here we tested whether CetZ2 influences cell shape in stationary phase compared to mid-log phase using deletion, overexpression, and GTPase mutations. For the following experiments, steady mid-log cultures of these strains were first diluted to a theoretical OD_600_ of 0.0005 in 10 mL Hv-YPCab medium with 0.2 or 1 mM ʟ-tryptophan (Trp) to induce expression of the relevant plasmid-encoded genes under the control of the p.*tna* promoter (25). Samples for mid-log, early stationary, mid-stationary, and late stationary phases were then taken after 24, 72, 96, and 120 h of further incubation, respectively (see Fig. 1A).

**Fig 2:**
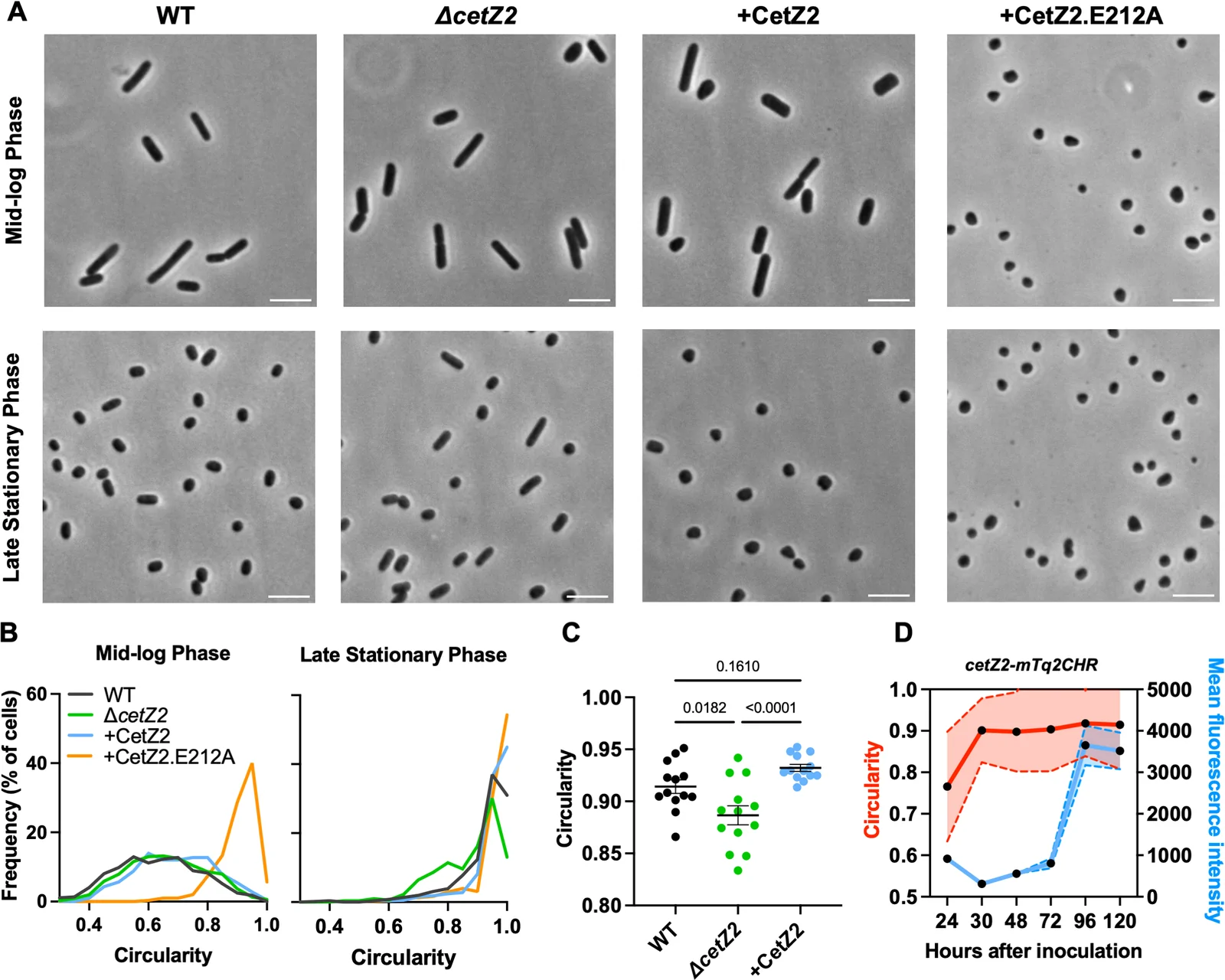
CetZ2 contributes to the maintenance of plate shape in stationary phase*.* (**A**) H26 + pTA962 (WT), Δ*cetZ2* + pTA962, Δ*cetZ2* + pTA962-CetZ2, and WT + pTA962-CetZ2.E212A were grown in Hv-YPCab medium supplemented with 1 mM ʟ-Trp and sampled during mid-log phase (24 h after inoculation) and late stationary phase (120 h after inoculation). Phase-contrast microscopy images (**A**) (scale bar = 5 µm) and (**B**) circularity analysis of individual cells during mid-log and late stationary phase. One representative culture replicate for each strain is shown. (**C**) Mean cell circularity (SD) of 13 replicate cultures in late stationary phase (120 h). One-way analysis of variance was used as a statistical test. The total number of cells analyzed from the 13 culture replicates was: WT (+pTA962), *n* = 14,242; Δ*cetZ2* (+pTA962), *n* = 23,671; Δ*cetZ2* (+pTA962-cetZ2), *n* = 16,416. (**D**) *cetZ2-mTq2CHR* + pTA962 was grown in Hv-YPCab medium from a theoretical starting OD_600_ ~0.0005 and observed by phase-contrast and fluorescence microscopy after 24, 30, 48, 72, 96, and 120 h of culturing. The circularity and mean fluorescence intensity of individual cells were analyzed at each time point. Individual points represent the mean of the population at each timepoint, and dotted lines indicate standard deviation. Two culture replicates were conducted, and the number of cells analyzed at each timepoint is as follows: 24 h, *n* = 253; 30 h, *n* = 554; 48 h, *n* = 1,209; 72 h, *n* = 1,872; 96 h, *n* = 728; 120 h, *n* = 187.

We initially compared cell shape phenotypes at the mid-log and late-stationary phases. At the 24 h mid-log stage, where wild-type cells were largely in a rod-shaped form before reverting to plate-shaped cells, deletion or overexpression of *cetZ2* had no obvious effect on cell shape, whereas production of the GTPase inactive mutant, CetZ2.E212A, strongly disrupted rod development as expected (1, 21) (Fig. 2A, top panels). These observations were quantified and confirmed by analysis of the circularity of cell outlines (Fig. 2B, left). In late stationary phase (120 h), the wild-type strain showed a mixture of mainly plate cells with some short rod shapes (Fig. 2A, lower panels). However, deletion of *cetZ2* resulted in more cell elongation in late stationary phase, whereas overexpression of *cetZ2* or *cetZ2*.E212A increased cell circularity in comparison to the wild type (Fig. 2A and C). These phenotypes were clear with multiple culture replicates (Fig. 2C) and demonstrate that CetZ2 has a role in maintaining plate shape in stationary phase.

To investigate the relative timing of CetZ2 upregulation and the transition to plate shape that occurs in mid-late log growth (18, 19), we created a chromosomal fusion of *cetZ2* to a mTurquoise2 fluorescent reporter, thus leaving *cetZ2* under the control of its native promoter (*cetZ2-mTq2CHR*). This strain was equivalent to the wild type in cell circularity (Fig. S2A) and CetZ2 upregulation in stationary phase (Fig. S1B), suggesting that the fusion is functional in the maintenance of plate shape. The total cellular fluorescence intensity of *cetZ2-mTq2CHR* and cell circularity were then measured during the transition to stationary phase (Fig. S1C). This showed that CetZ2 upregulation occurred between 72 h and 96 h of culturing (Fig. 2D, blue), which was clearly significantly later than the completion of the transition of rod-shaped cells to plate-shaped cells at approximately 30 h (Fig. 2D, red). This suggested that CetZ2 upregulation is not linked to mid-log plate shape transitions and instead has a role in the maintenance of plate shape in the stationary phase.

Previous work showed that *cetZ2* is dispensable for normal swimming motility in soft agar (1), yet its overexpression slightly enhanced motility (21). It was also known that the actively swimming cells at the leading edge of the expanding halo in soft agar are rod shaped, whereas those in the interior of the halo (that are slower or not swimming) are plate shaped (1). We therefore investigated the relative expression of *cetZ2* in these cells, using the reporter fusion strain. We found that the *cetZ2-mTq2CHR* strain was fully functional in swimming motility (Fig. S1D and E). Fluorescence measurements indicated that CetZ2-mTq2 fluorescence was very faint, and only detected above background in plate-shaped cells sampled from the inner motility halo (Fig. S1F and I). Thus, the relationship between relative CetZ2-mTq2 expression and cell morphology is consistent between batch culture and motility assay conditions. These observations are consistent with the notion that CetZ2 is specifically produced or effective in plate-shaped cells, which have already reverted from rod shaped (21).

### Overproduction of tagged CetZ2 reveals changes in subcellular localization during the progression through stationary phase

We observed that the *cetZ2-mTq2CHR* strain showed only extremely faint CetZ2-mTq2 subcellular localization relative to the cellular background during stationary phase (Fig. S2C). This was likely a combination of the naturally low concentration of CetZ2 in cells (Fig. 1F) and the moderate blue auto-fluorescence of *H. volcanii* cells. We then decided to overproduce CetZ2-mTq2 or a CetZ2-YPet fusion from a plasmid to achieve a greater signal-to-background ratio and investigate the effects of their overproduction compared to untagged CetZ2 during the progression through stationary phase.

We initially found that overproduction of CetZ2-mTq2 or CetZ2-YPet prevented rod formation in late stationary phase, equivalently to the overproduction of the untagged CetZ2 (Fig. S3A). Also, the inhibitory point mutants CetZ2.E212A-mTq2 and CetZ2.E212A-YPet had equivalent effects to untagged CetZ2.E212A in preventing rod formation (Fig. S3B). Western blotting confirmed strong overproduction of the tagged and untagged CetZ2 and CetZ2.E212A in these strains, compared to the low concentration of endogenous CetZ2 in the wild type (Fig. S3E), even without additional inducer, L-Trp (Fig. S3F). We also monitored CetZ2-mTq2 overproduction over time and saw that it increased further as stationary phase progressed (Fig. S3G). These results further suggest that the tagged CetZ2 proteins are functional and their overproduction leads to an accentuation of the prevention of rod formation in stationary phase.

**Fig 3:**
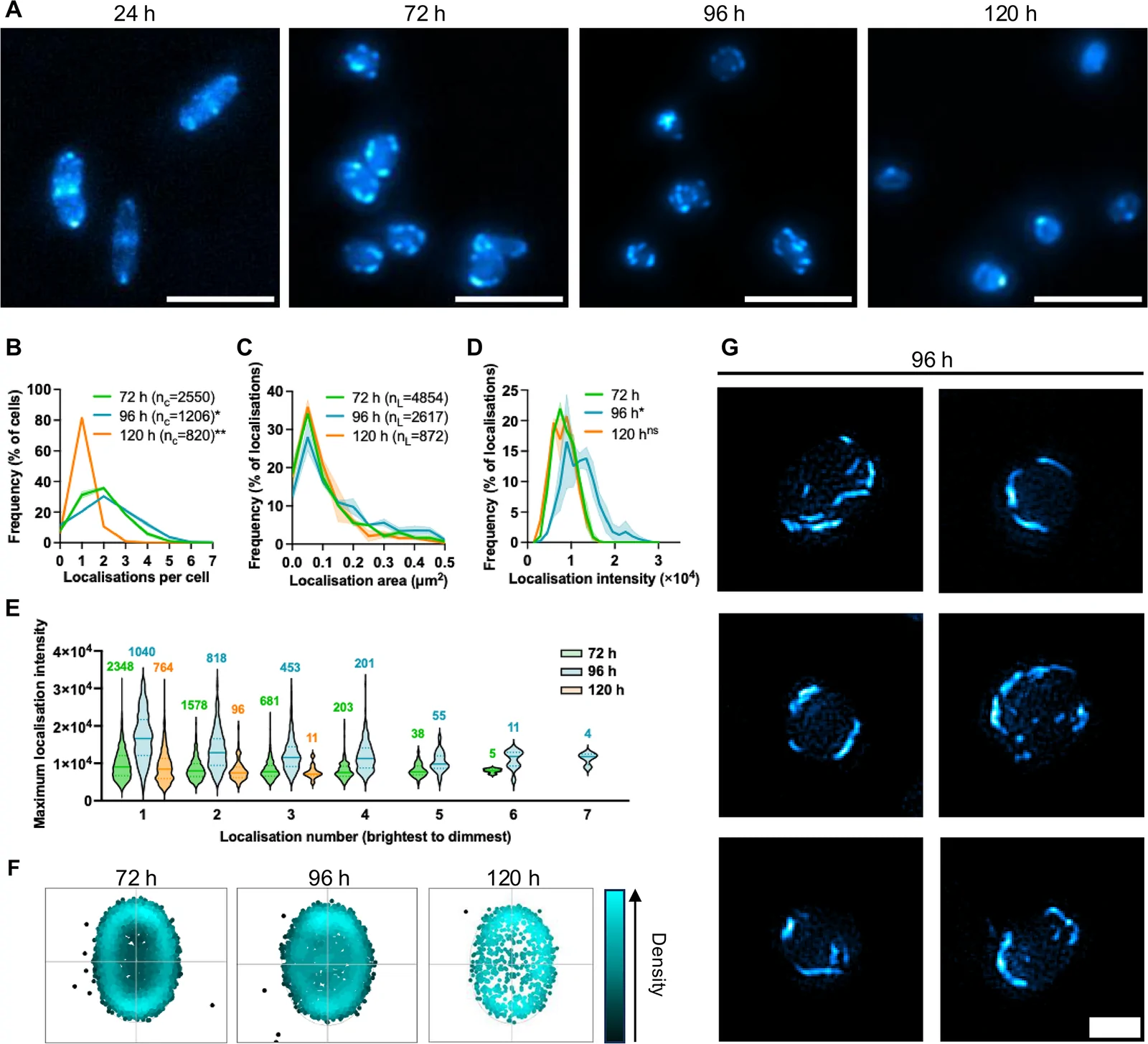
Subcellular localization of overproduced CetZ2-mTq2 in stationary phase. (**A**) WT + pHJB6 (for overproduction of CetZ2-mTq2) was grown in Hv-YPCab medium supplemented with 0.2 mM L-Trp and imaged using fluorescence microscopy at 24, 72, 96, and 120 h. Scale bar = 5 µm. Images are brightness and contrast adjusted for viewability of CetZ2-mTq2 localization characteristics. (**B**) Frequency distributions showing the number of localizations per cell. (**C**) Localization area and (**D**) mean localization intensity (the sum of pixel values, normalized by the number of pixel values within the boundary of a detected localization) in all localizations detected in all cells. (**E**) Maximum fluorescence intensity distributions separated by their relative intensity per cell, comparing 72, 96, and 120 h. The number of localizations constituting each violin plot is detailed above it. (**F**) Heatmap representations of CetZ2-mTq2 localizations, colored by density to indicate the frequency of localization. Two independent replicates were carried out for panels **A–F**, the total number of cells analyzed (*n*_*c*_) and the total number of localizations detected from those cells (*n*_*L*_) at each timepoint are indicated in the key in panels **B** and **C**, respectively, and apply to panels **B–F**. (**G**) 3D structured-illumination microscopy maximum intensity Z-projections of CetZ2-mTq2 at 96 h. Scale bar = 2 µm. In panels **B–D**, the bold line indicates the mean of independent replicates, and the shaded area indicates standard error. One-way analysis of variance was used to compare the means of culture replicates at 96 h or 120 h to 72 h, and results are indicated in the keys of panels **B–D** (ns = not significant, *P < 0.02 and **P < 0.002).

We next used fluorescence microscopy to investigate the subcellular localization of the overproduced CetZ2-mTq2 (Fig. 3A). During the mid-log phase (24 h), CetZ2-mTq2 localized in patches throughout the cell, around the membrane, at mid-cell, and at the cell poles, similar to the previously reported localization of CetZ1 in mid-log cultures (1, 23) and consistent with the recently reported localization of overproduced CetZ2 in motile rod-shaped cells (21). Similarly, in early (72 h) and mid (96 h) stationary phase, the overproduced CetZ2-mTq2 localized mostly as foci, patches, or short filaments around the edges of the plate-shaped cells, whereas late stationary phase (120 h) cells showed noticeably fewer distinct foci (Fig. 3A and F). This was confirmed by quantitative analysis of the localization frequency per cell (Fig. 3B). There were no significant differences in the cell size (area), cell shape (circularity), or the size (area) of the CetZ2-mTq2 localizations among all three stationary phase timepoints (Fig. 3C and Fig. S3C and D). Thus, the frequency of localizations per unit of cell area did not change significantly between early and mid-stationary phases, but in the late stationary phase (120 h), the CetZ2-mTq2 localizations were less frequent, and they also decreased in area and maximum intensity in comparison to early and mid-stationary phases (Fig. 3B through D). Interestingly, the fluorescence intensity of localizations increased reproducibly at 96 h compared to 72 h and 120 h (Fig. 3D and E), suggesting a potentially greater activity or clustering/assembly of CetZ2 specifically at mid-stationary phase. These findings suggest that progressive changes in CetZ2 activities occur during the stationary phase, with the greatest apparent localization activity occurring in mid-stationary phase.

To visualize the mid-stationary-phase assemblies at higher resolution, we also used 3D structured-illumination microscopy (3D-SIM) at 96 h, which revealed clear filaments or patches around the edges of the cell that were infrequently seen on the top or bottom surfaces (Fig. 3G and Video S1).

To investigate potential impacts of the CetZ2-mTq2 overproduction on the patterns observed, we compared cells induced with 0, 0.05, 0.1, 0.2, or 1 mM L-Trp. The four lower concentrations (0–0.2 mM L-Trp) did not result in substantial differences in the observed localization pattern (Fig. S4A), the number of localizations per cell (Fig. S4C), or the localization area (Fig. S4D). Although, as expected, the mean intensity of localizations increased with the increasing amounts of L-Trp (Fig. S4E). Significant changes in the localization pattern were only observed with the highest concentration of L-Trp (1 mM), where some cells showed bright clusters of CetZ2-mTq2 (Fig. S4A, D, and E), which are likely artifacts of the strong overproduction. Given that the CetZ2-mTq2 patterns appeared the same within a range of the lower overproduction levels that also promote plate cell shape maintenance, they likely reflect functionally relevant structures of CetZ2.

### Subcellular structures containing overproduced CetZ2-mTq2 undergo dynamic remodeling in mid-stationary phase

**Fig 4:**
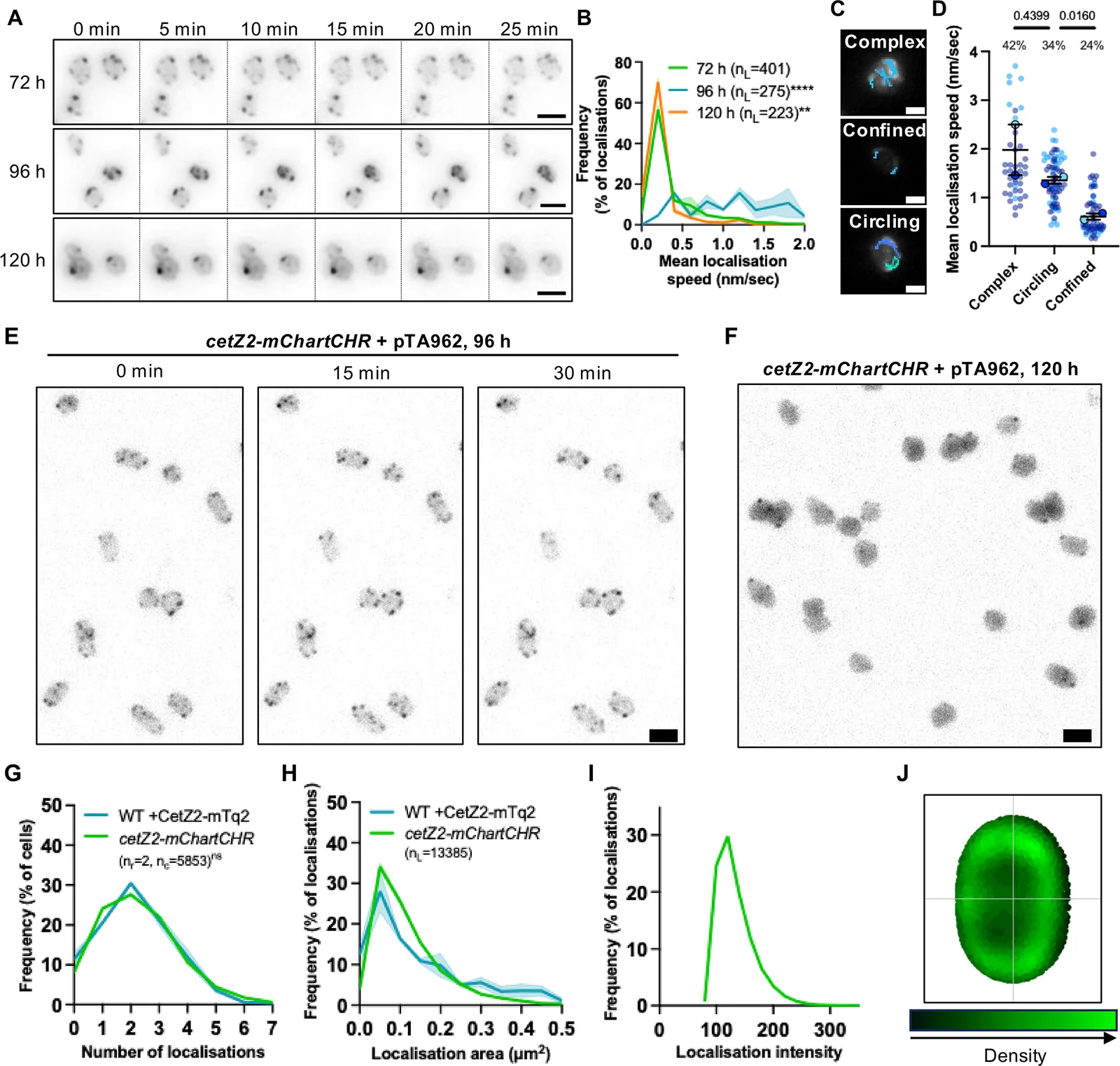
Localization dynamics of CetZ2 in stationary phase. (**A**) Time-lapse imaging of overproduced CetZ2-mTq2 at 72, 96, and 120 h. Images were taken at 1 min intervals for 30 min. Scale bar = 2 µm. TrackMate2 was used to track localization speed of *XY*-projected motion of overproduced CetZ2-mTq2 only. The analysis does not take the *Z*-plane into account and therefore may be an underestimation of true speed in three dimensions. (**B**) Frequency distribution of localization speed. Two independent replicates were carried out, and the total number of detected localizations (*n*_*L*_) at each timepoint is indicated in the key. The bold line indicates the mean of independent replicates, and the shaded area indicates the standard error. (**C**) Overlayed tracks of localization dynamics generated in TrackMate2 were used to define characteristic patterns of overproduced CetZ2 dynamics in mid-stationary phase (96 h). Scale bar = 1 µm. (**D**) Mean localization speed by dynamic pattern as defined in panel **C.** The total number of cells categorized was 176 (pooled from two independent replicates, represented in different colors), and the percentage of cells assigned to each dynamic pattern is indicated above each category. Individual points represent individual cells; large data points indicate the mean of each culture replicate. The mean and standard error are shown. (**E**) *cetZ2-mChartCHR* + pTA962, which produces CetZ2-mChart under the control of the native *cetZ2* promoter, was grown in Hv-YPCab medium and imaged three times, with 15-minute intervals, during mid-stationary phase (96 h) (Video S4). (**F**) CetZ2-mChart localization during late stationary phase (120 h). Images are brightness and contrast adjusted for viewability. Localization analysis was carried out for cells sampled in mid-stationary phase (96 h). Frequency distributions showing (**G**) the number of localizations per cell, (**H**) localization area, and (**I**) mean localization intensity (the sum of pixel values, normalized by the number of pixel values within the boundary of a detected localization). (**J**) Heatmap representation of CetZ2-mChart localization, colored by density to indicate frequency of localization. Four independent replicates of imaging were conducted, and two (*n*_*r*_) were used for image analysis in panels **G–J**. The number of cells (*n*_*c*_) and localizations (*n*_*L*_) pooled and analyzed are indicated in panels** G** and **H**, respectively, and apply to panels **I** and **J**. Data for WT cells overproducing CetZ2-mTq2 from a plasmid in panels **G** and **H** are duplicated from Fig. 3B and C, respectively, for comparison. In panels **B** and **G–I**, the bold line indicates the mean of independent replicates, and the shaded area indicates the standard error. One-way analysis of variance was used in panels **B** (comparing 96 and 120 h to 72 h) and **D**, and an unpaired *t*-test was used in panels **G** and **H** to compare the means of culture replicates. Results are indicated in each panel (ns = not significant, **P < 0.002 and ****P < 0.0001).

We next sought to investigate the potential dynamic behavior of the assemblies containing CetZ2-mTq2. To observe any short-term dynamic movement of the overproduced CetZ2-mTq2, we used time-lapse imaging at the main stages of stationary phase. In early and late stationary phase cells, imaged over 30 min at 1 min intervals, CetZ2-mTq2 localization appeared relatively static, but, remarkably, in mid-stationary phase it was relatively dynamic (Fig. 4A and Video S2). Computational tracking of the apparent filaments, patches, and foci (Video S3) confirmed that movement of these structures was fastest in mid-stationary phase (Fig. 4B). At this stage, cells displayed three main types of dynamic CetZ2-mTq2 patterns (Fig. 4C and Video S2). These included (i) a “circling” pattern (42% of cells), in which CetZ2-mTq2 was observed traveling around the perimeter of the cell in a manner consistent with treadmilling, (ii) a complex pattern (24% of cells), which showed an apparently more random yet directional motion within the cell, and (iii) a “confined” pattern (34% of cells), which were static or relatively slow and envelope associated. Trajectories displaying the complex dynamics were fastest, followed by circling, and then confined patterns (Fig. 4D). Equivalent dynamic behaviors in mid-stationary phase (96 h) were still observed when CetZ2-mTq2 production was reduced by withholding additional L-Trp from the media (Fig. S4F) or in the cells that did not have the large bright clusters when 1 mM L-Trp was used (Fig. S4F).

### Dynamic localization of tagged CetZ2 produced from its native chromosomal locus

While the overproduced CetZ2-mTq2 was dynamic and functional in promoting plate cell maintenance, this condition might not perfectly reflect CetZ2 at its native concentration. To address this, we generated *cetZ2* chromosomal fusions with newly available brighter fluorescent proteins, mChartreuse and mLemon (*cetZ2-mChartCHR* and *cetZ2-mLemCHR*). Both strains exhibited the expected upregulation of CetZ2 concentrations in stationary phase and were significantly brighter than *cetZ2-mTq2CHR* (Fig. S5A and S5B). They also exhibited equivalent cell circularity to wild type and *cetZ2-mTq2CHR* during mid-stationary phase (Fig. S5D).

We focused on *cetZ2-mChartCHR* and found it displayed similar localization patterns to the overproduced CetZ2-mTq2 in both mid- and late-stationary phases, including the expected foci and short patches or filaments that diminished in late stationary phase (Fig. 4E and F). As expected, the localization intensity of CetZ2-mChart was much lower than during overexpression of *cetZ2-mTq2* (Fig. 4I and 3D), although there was a similar number of localizations per cell (Fig. 4G). CetZ2-mChart did not appear to produce quite as many of the relatively large patch or filament-like structures as observed in some cells overproducing CetZ2-mTq2 (Fig. 4E and 3A), and this was reflected in the quantification of localization area (Fig. 4H), where somewhat fewer relatively large assemblies and more of the small single foci (diffraction-limited points) can be seen.

We were unable to acquire sufficient time-lapse imaging data for quantitative tracking of foci, which we attributed to the very low level of native CetZ2 and photobleaching in our current imaging system. However, we were able to detect CetZ2-mChart with three images at 15 min intervals and qualitatively detected mid-stationary phase movement with *cetZ2-mChartCHR* (Fig. 4E and Video S4) or *cetZ2-mLemCHR* (Fig. S5B and Video S4). Given the similarities in localization patterns, dynamics, and functionality of the native-level and overproduced CetZ2-FPs, we conclude that their common behaviors reflect functionally relevant activities of CetZ2. Furthermore, the relatively highly dynamic activities of CetZ2 specifically in mid-stationary phase strongly suggest that CetZ2 is regulated by other molecules or factors at this stage.

### Mid-stationary phase CetZ2 dynamic subcellular structures are dependent on the presence of CetZ1 and on the GTPase active site of CetZ2

**Fig 5:**
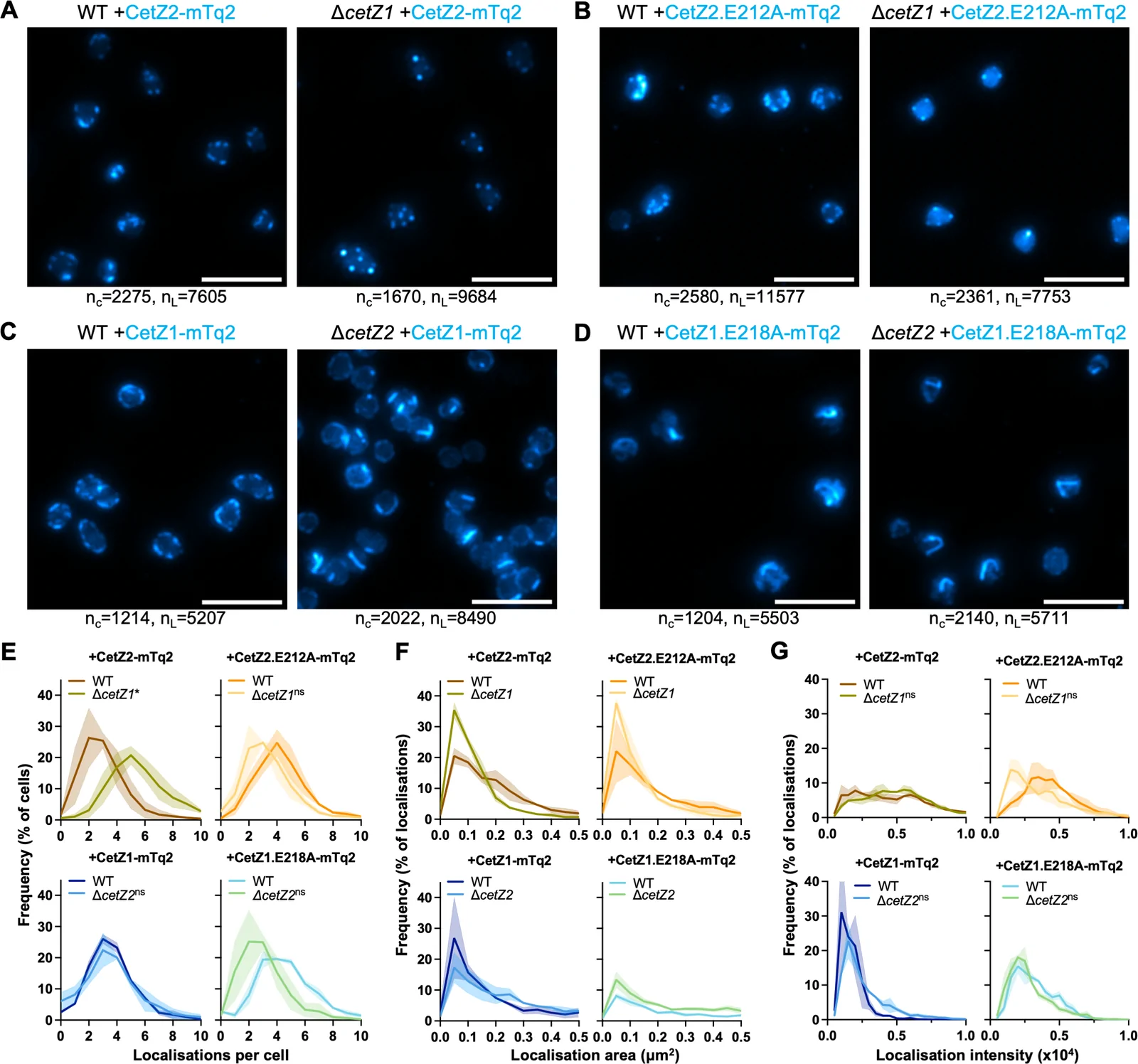
Localization interdependency of CetZ1 and CetZ2 in mid-stationary phase. Cells were grown in Hv-YPCab medium supplemented with 0.2 mM ʟ-Trp for overproduction of fusion proteins and imaged after 96 h (mid-stationary phase). (**A**) CetZ2-mTq2 (pHJB6) and (**B**) CetZ2.E212A (pHJB12) localization in WT and Δ*cetZ1*. (**C**) CetZ1-mTq2 (pHVID135) and (**D**) CetZ1.E218A-mTq2 (pHJB13) localization in WT and Δ*cetZ2*. For panels **A–D**, scale bar = 5 µm and images are brightness and contrast adjusted for viewability. Localization analysis was carried out on this data, measuring (**E**) the number of localizations per cell, (**F**) mean intensity of localizations (the sum of pixel values, normalized by the number of pixel values within the boundary of a detected localization), and (**G**) the distance of each localization from the cell edge. Three independent replicates were carried out. The number of cells (*n*_*c*_) and localizations (*n*_*L*_) pooled from replicates is indicated below the images in panels **A–D** and applies to panels **E–G**. In panels **E–G**, the bold line indicates the mean of independent replicates, shaded area indicates standard error. One-way analysis of variance was used as a statistical test to compare the means of culture replicates of wild type and Δ*cetZ1* or Δ*cetZ2* backgrounds. Results are indicated in the keys of each panel (ns = not significant, *P < 0.02).

As CetZ2 is only present in species that also have CetZ1 (17), and CetZ2.E212A production inhibits CetZ1-dependent motile rod development (1), it might normally maintain plate shape in stationary phase by inhibiting or counteracting CetZ1 function. To begin investigating such potential interactions, we determined whether each protein’s subcellular localization in mid-stationary phase was dependent on the presence of the other one. Interestingly, in the absence of CetZ1 (Δ*cetZ1* background), the overproduced CetZ2-mTq2 did not form substantial patches or filaments as seen in the wild-type background, and instead it mislocalized as immobile single foci (Fig. 5A and Video S5); analysis confirmed that while the average number of CetZ2-mTq2 localizations per cell increased, the localization area decreased to a size distribution consistent with diffraction-limited foci (Fig. 5E and F). These results suggest that the appearance of substantial dynamic subcellular assemblies containing CetZ2 in mid-stationary phase is strongly dependent on the presence of CetZ1.

To see if the expected GTPase activity affects CetZ2’s behavior, we investigated the localization and movement of CetZ2.E212A-mTq2 in stationary phase. The localization patterns of this mutant showed some similarities to CetZ2-mTq2 but included brighter and larger assemblies (Fig. 5A and B). Furthermore, the overall patterns appeared largely unchanged throughout stationary phase (Fig. S7A), and, in 30 min movies, CetZ2.E212A-mTq2 was strikingly non-dynamic compared to CetZ2-mTq2 (Fig. S7B and Video S6). In the absence of CetZ1, CetZ2.E212A-mTq2 was also non-dynamic, as expected (Video S5), but showed fewer foci per cell and greater diffuse cellular fluorescence when compared to the wild-type background (Fig. 5B, E, and G). These results indicate that the dynamics of CetZ2-mTq2 structures in mid-stationary phase are dependent on its GTPase activity and the expected subunit disassembly and turnover it enables. Furthermore, despite the high stability of the CetZ2.E212A-mTq2 assemblies, CetZ1 is still required for the formation of their relatively large subcellular structures.

To investigate whether the effects of CetZ1 or the CetZ2 GTPase active site are stationary-phase specific, we also looked at samples from mid-log phase. CetZ2-mTq2 mid-log localization patterns generally appeared similar to those of CetZ1-mTq2 (Fig. S8A and C). In mid-log, the patches of CetZ2-mTq2 were noticeably more distributed throughout the cell area compared to the prevalent cell-edge patches seen during stationary phase (Fig. 3A and G and 5A). The mid-log localization of CetZ2-mTq2 and CetZ2.E212A-mTq2 was also reduced in the absence of CetZ1 compared to wild type (Fig. S8A and C). These results suggest that CetZ1 influences CetZ2 localization in both log and stationary phase, and other stationary-phase-specific factors also influence the positioning of CetZ2.

### CetZ2 influences the formation and stability of CetZ1 subcellular structures in stationary phase

We next investigated the influence of stationary phase and CetZ2 on the *in vivo* assembly of CetZ1. In the wild-type background in mid-stationary phase, CetZ1-mTq2 localized in patches and small foci, mostly concentrated around the cell edge, but occasionally, larger apparent filaments were also observed (Fig. 5C). Time-lapse imaging showed that while some of the small foci and diffuse regions of CetZ1-mTq2 localization appeared dynamic, the substantive filaments and patches were relatively stable over the 30 min (Video S7). This contrasts the dynamic behavior of CetZ1 in mid-log where it is active in rod cell development (1, 23), suggesting that stabilization or inhibition of CetZ1 occurs during stationary phase.

We then looked at the effects of CetZ2 in mid-stationary phase and saw that CetZ1-mTq2 localization patterns appeared generally similar in the wild type and Δ*cetZ2* backgrounds, though, without *cetZ2*, there were more of the relatively large brighter filaments (Fig. 5C and F). Strikingly, in the absence of CetZ2, some of the bright CetZ1 filaments were clearly seen to shrink over time, which was not observed in the wild-type background (Video S7). This would suggest that CetZ2 can both limit CetZ1 assembly and stabilize existing CetZ1 filaments in stationary phase.

The CetZ1 GTPase mutant (CetZ1.E218A) is known to distort cell shape and hyperstabilize filaments, which are expected to contain GTP-bound polymers that are incapable of GTP hydrolysis-based disassembly (1). Here, in mid-stationary phase, we found that CetZ1.E218A-mTq2 showed similar patterns and appeared as substantive, often curved filaments in both the wild type and Δ*cetZ2* backgrounds (Fig. 5D), although somewhat less frequently in the absence of CetZ2 (Fig. 5E). The CetZ1.E218A-mTq2 assemblies were not dynamic in both wild type and Δ*cetZ2* backgrounds during the 30 min time-lapse imaging (Video S7). During mid-log phase, CetZ1-mTq2 and CetZ1.E218A-mTq2 showed their expected localization patterns based on previous data (1), which appeared unaffected by the absence of CetZ2 (Fig. S8B) as may be expected given the very low concentration of CetZ2 in mid-log (Fig. 1). Thus, CetZ2 does not appear to substantially destabilize the CetZ1.E218A assemblies.

### *cetZ1* or *cetZ2* GTPase mutants interfere with the localization dynamics of both wild-type proteins

Given the genetic interactions between *cetZ1* and *cetZ2* detected above, we investigated the effect of each GTPase mutant on the localization and dynamics of the other CetZ in mid-stationary phase. We created four plasmids for dual expression of *cetZ2 + cetZ1-mTq2*, *cetZ2*.E212A *+ cetZ1-mTq2*, *cetZ2-mTq2 + cetZ1*, and *cetZ2-mTq2 + cetZ1*.E218A in a bicistronic configuration, respectively, under the control of the p.tna promoter.

**Fig 6:**
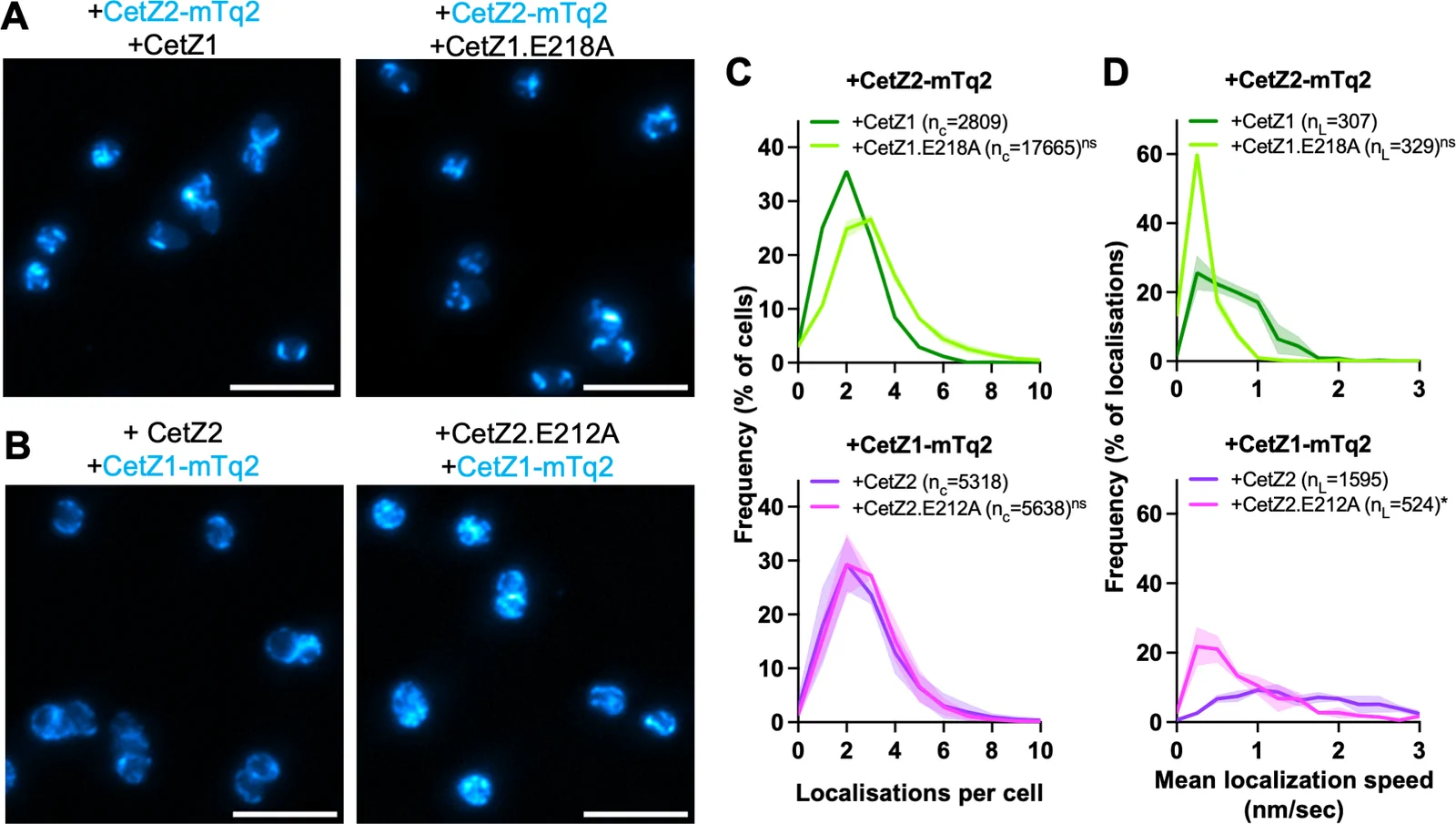
GTPase mutations in CetZ1 or CetZ2 disrupt the localization and dynamics of the other CetZ in mid-stationary phase. (**A**) CetZ2-mTq2 was overproduced with untagged CetZ1 (pHJB45) or untagged CetZ1.E218A (pHJB46) in the WT background. (**B**) CetZ1-mTq2 was overproduced with untagged CetZ2 (pHJB43) or untagged CetZ2.E212A (pHJB44) in the WT background. Scale bar = 5 µm. Images are brightness and contrast adjusted for viewability. Localization analysis was conducted on data from panels **A** and **B**, measuring (**C**) the number of localizations per cell. For panels **A–C**, three independent replicates were carried out, and the number of cells (*n*_*c*_) and localizations (*n*_*L*_) pooled from replicates is indicated in the keys of panel panel **C**. (**D**) Time-lapse imaging was conducted for strains in panels **A** and **B** (Video S8), and the mean localization speed for each focus was measured. The number of localizations (*n*_*L*_) measured from two independent replicates is indicated in the key. In panels **C** and **D**, the bold line indicates the mean of independent replicates, and the shaded area indicates standard error. One-way analysis of variance was used as a statistical test to compare the means of culture replicates of the +CetZ1 and +CetZ1.E218A backgrounds, or the + CetZ2 and +CetZ2.E212A backgrounds. Results are indicated in the key of each panel (ns = not significant, *P < 0.02).

The patterns of CetZ1-mTq2 localization during co-expression with either *cetZ2* or *cetZ2*.E212A had a similar appearance and number of localizations per cell (Fig. 6B and C). However, time-lapse imaging and tracking of focus speed revealed that co-expression with *cetZ2*.E212A partially stabilized the CetZ1-mTq2 assemblies compared to co-expression with *cetZ2* (Video S8 and Fig. 6D). Likewise, the patterns of CetZ2-mTq2 localization were also similar during co-expression with *cetZ1* or *cetZ1*.E218A (Fig. 6A and C), and the mean focus speed of CetZ2-mTq2 was reduced by co-expression of *cetZ1*.E218A compared to *cetZ1* (Video S8 and Fig. 6D). These results suggest that the GTPase activity of CetZ1 or CetZ2 has a significant impact on the assembly characteristics of both proteins, further suggesting that these proteins genetically (and potentially physically) interact to control cell morphology in mid-stationary phase.

### CetZ1 and CetZ2 localization is partially correlated in mid-stationary phase

The above results suggested that CetZ1 and CetZ2 polymerization-like behaviors are at least partially interdependent in stationary phase, consistent with the view that their functions are interlinked, with an antagonistic activity of CetZ2 against the rod development function of CetZ1. This prompted us to determine whether CetZ1 and CetZ2 can colocalize and to observe their dynamics in relation to one another during mid-stationary phase.

**Fig 7:**
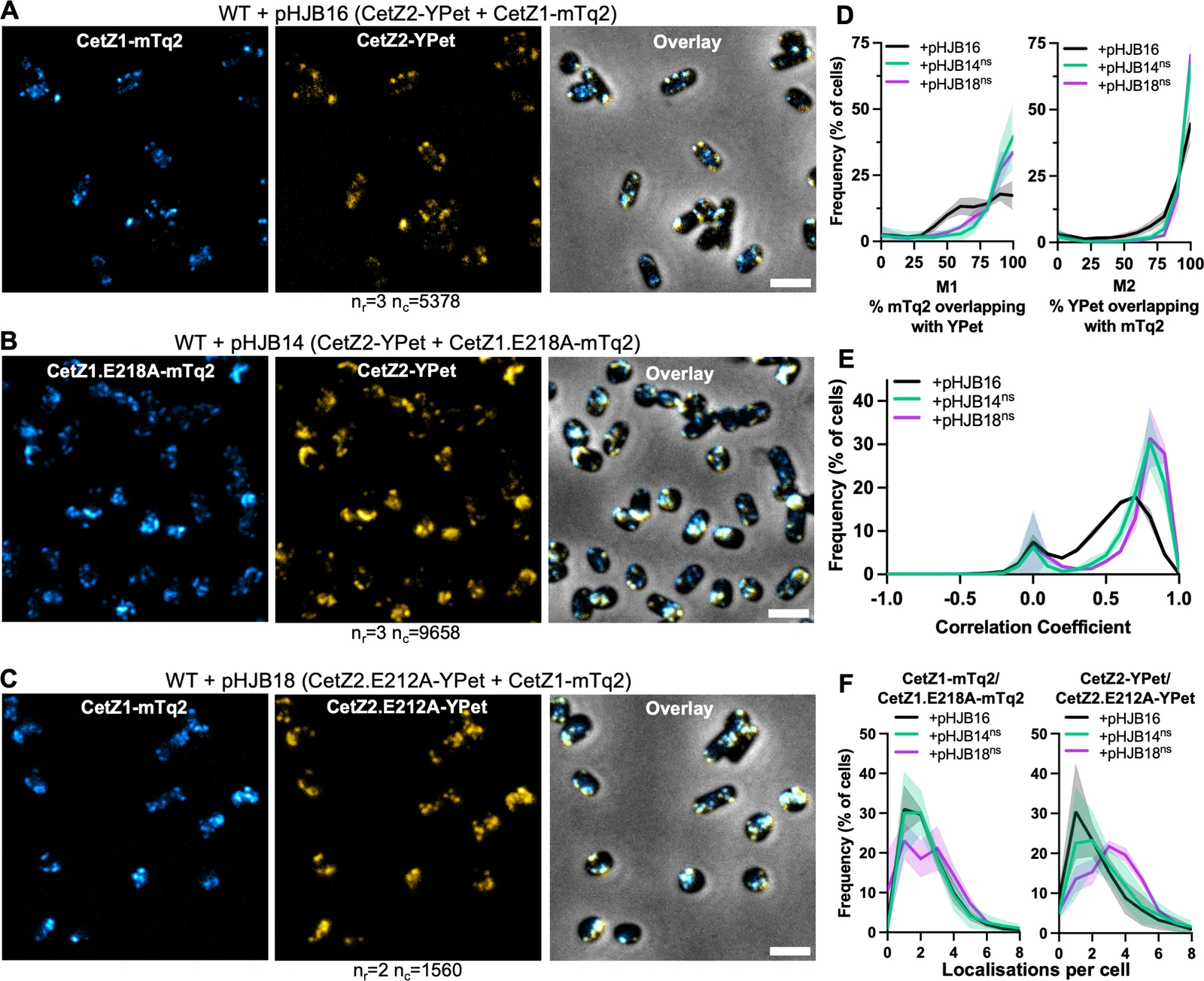
Co-production and localization of overproduced CetZ1 and CetZ2 fluorescent protein fusions in mid-stationary phase. Wild-type cells overproducing CetZ1-mTq2 and CetZ2-YPet (pHJB16), CetZ1.E218A-mTq2 and CetZ2-YPet (pHJB14), or CetZ1-mTq2 and CetZ2.E212A-YPet (pHJB18) were grown in Hv-YPCab medium supplemented with 0.05 mM ʟ-Trp to induce expression of fluorescent proteins while avoiding artefactual aggregations apparent at high induction levels. (**A**) WT + pHJB16, (**B**) WT + pHJB14, and (**C**) WT + pHJB18 were visualized using phase contrast and fluorescence microscopy during mid-stationary phase. Scale bars = 2.5 µm and apply to all panels. Images are brightness and contrast adjusted for viewability. The number of independent replicates (*n*_*r*_) and analyzed cells (*n*_*c*_) per strain are indicated in panels **A–C** and apply to panels **D–F**. Images of each strain were analyzed using EzColocalization. Distributions showing (**D**) the proportion of mTq2 fluorescent signal overlapping with YPet fluorescent signal (M1) and the reciprocal (M2). (**E**) Pearson correlation coefficients of CetZ1/CetZ1.E218A-mTq2 and CetZ2/CetZ2.E212A-YPet fluorescence per cell, represented as a distribution. A small peak at 0 was observed for all strains, resulting from a small proportion of cells that only displayed fluorescence in one channel, or no fluorescence. (**F**) The number of CetZ1/CetZ1.E218A-mTq2 and CetZ2/CetZ2.E212A-YPet localizations per cell were counted for each strain. In panels D–F, the bold line indicates the mean of independent replicates, and the shaded area indicates standard error. One-way analysis of variance was used as a statistical test to compare the means of culture replicates of WT + pHJB14 and WT + pHJB18 to WT + pHJB16. Results are indicated in the key of each panel (ns = not significant).

When *cetZ1-*mTq2 and *cetZ2-*YPet were co-expressed in the wild-type background, both proteins displayed patchy localizations that appeared mostly around the edges of the cells (Fig. 7A). Co-localization analysis showed that CetZ1-mTq2 and CetZ2-YPet localizations imperfectly overlapped, and the proportion of CetZ2-YPet overlapping with CetZ1-mTq2 (M2, mean of 82%) was somewhat higher than the inverse relationship (M1, mean of 70%; Fig. 7D), suggesting that there are slightly more “free” localizations of CetZ1-mTq2 than CetZ2-YPet in this overexpression experiment. Pearson correlation analysis showed that 36% of cells had a co-localization correlation coefficient ≥0.7 (Fig. 7E), further demonstrating their partial overlap. This partial correlation between CetZ1 and CetZ2 localizations is consistent with their differing dynamics, where CetZ2 is highly dynamic but CetZ1 is not (Fig. 3H through K, 4A, and 6E), and indicates CetZ1 and CetZ2 localizations tend to be associated with one another but are not obligately co-localized. It should be noted that the overexpression necessary to sufficiently visualize the proteins in these experiments may not perfectly reflect the degree of co-localization in wild-type cells.

We then co-expressed the two tagged proteins, where one was the hyperstable GTPase mutant, to assess whether this would result in greater co-assembly (Fig. 7B and C). The CetZ1.E218A-mTq2 overlap with CetZ2-YPet (M1) increased to a mean of 83% while the CetZ2-YPet overlap with CetZ1.E218A-mTq2 (M2) increased to a mean of 91% (Fig. 7D), and ~70% of cells had a Pearson’s correlation coefficient ≥0.7 (Fig. 7E). Indistinguishable results were obtained with the alternate CetZ2.E212A-YPet and CetZ1-mTq2 combination (Fig. 7D and E). Finally, the number of foci per cell moderately increased in the strain producing CetZ1-mTq2 and CetZ2.E212A-YPet compared to the dual wild-type proteins (Fig. 7F). In summary, both combinations with the GTPase mutations showed a substantial increase in the degree of co-localization compared to the two wild-type proteins, demonstrating an interdependency between the GTPase functions of the two proteins and direct or indirect co-assembly.

## DISCUSSION

In this study, we describe the function of archaeal tubulin-like protein CetZ2 and its interplay with its paralog CetZ1 that leads to the coordination of shape control and pleomorphism in the growth cycle of the model archaeon *H. volcanii*. In early log phase growth, *H. volcanii* cells largely transition from the plate (or disk-like) cells into rod-shaped cells, until later in log phase when they revert into plates and are maintained as such in stationary phase (1, 18). CetZ1 is necessary for plate cells to develop into rods, and it does so independently of CetZ2 (1), which we found here is only produced at a very low level in these conditions. The mid-log reversion from rods to plates is mediated by several other factors, including DdfA and the cytoskeletal proteins volactin and halofilins HalA/HalB (26, 27). Here, we saw that CetZ2 does not have a clear role in rod development or reversion to plates, both of which occur well before its strong upregulation as cells enter stationary phase. CetZ1 was much more abundant than CetZ2 throughout the growth cycle, but it is moderately downregulated in stationary phase. The strong increase in the ratio of CetZ2 to CetZ1 as cells enter stationary phase correlated with the observation that CetZ2 is required to help maintain plate shape specifically in stationary phase.

We observed a peak of CetZ2 subcellular localization specifically in mid-stationary phase. By overproducing a fluorescent-tagged CetZ2, we observed clear filaments or patches around the edge of the cells that were noticeably larger and showed more dynamic remodeling in mid-stationary phase compared to other stages of culture. Although overproduction was necessary to properly track such movement, consistent observations were also made with a functional chromosomal fusion of CetZ2-mTq2 produced at its very low native concentration. Furthermore, the CetZ2 dynamic patterns were similar across a range of moderate overproduction conditions, and the tagged and untagged CetZ2 were indistinguishable in promoting the maintenance of plate cell shape. Together, these findings suggest that the patterns reflect biologically relevant structures even if they do not perfectly reflect the wild-type CetZ2 concentration. Secondly, they raise the possibility that CetZ2 might not act as a relatively simple self-assembled polymer that would grow larger with more protein present. Instead, it is likely to associate with other structures that primarily influence the size and shape of the patterns observed. Despite this, the movement of the tagged CetZ2 around the cell was clearly directional, displaying a circling-like behavior in some cases, which likely reflects protein polymer treadmilling and a cytomotive function (28) in CetZ2 or other polymers it may associate with. The structures and composition of these assemblies *in vivo*, and exactly how their behavior could lead to the maintenance of plate cell shape, thus remain to be determined.

**Fig 8:**
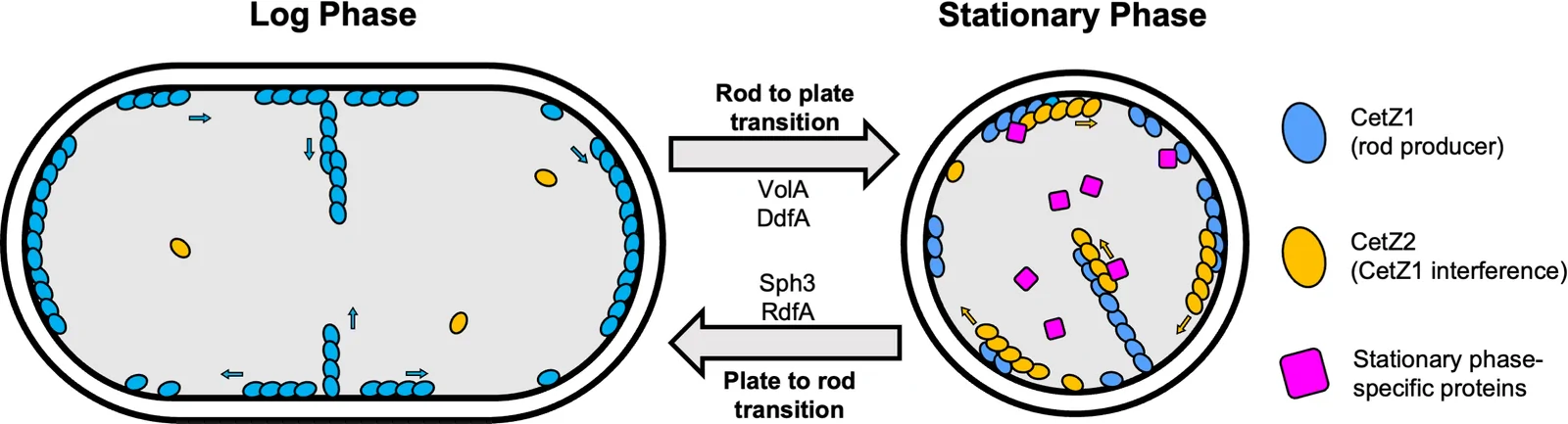
Model for the coordination of shape change by CetZ proteins in the *H. volcanii* growth cycle. In early and mid-log phase, CetZ1 is abundant and forms dynamic structures adjacent to the inner membrane at cell poles, mid-cell, and the long axis of cells (23), and is necessary for rod development with other factors such as RdfA and Sph3 (27). CetZ2 is at a very low level and not involved in either of the cell shape transitions in early/mid-log. The rod-to-plate cell transition in the mid-late log phase before cells enter stationary phase is likely controlled by a number of factors such as volactin (VolA) and DdfA (27). In stationary phase (right), CetZ2 is upregulated, and CetZ1 is downregulated, which might enable CetZ2 to counteract CetZ1 function, through direct or indirect interactions, thereby maintaining plate shape by preventing rod development in stationary phase.

The above findings were taken as the basis for investigating potential interplay between CetZ1 and CetZ2 functions, which suggested that CetZ2 contributes to plate shape maintenance in stationary phase by counteracting the CetZ1-based rod-development pathway (schematic in Fig. 8). This manifested as CetZ2-dependent effects on CetZ1 cytoskeletal structures and their dynamic behaviors in stationary-phase cells. For example, CetZ1 assemblies were more unstable in the absence of CetZ2 in mid-stationary phase. If CetZ2 directly or indirectly stabilizes CetZ1 polymers, this might inhibit CetZ1’s rod-development function, which relies on GTPase-dependent disassembly and dynamic turnover of its cytoskeletal structures (1). Secondly, we found that production of the CetZ2 GTPase mutant, which hyper-stabilizes the protein filaments, increased the extent of the observed colocalization of CetZ1 and CetZ2 in stationary phase, further suggesting that the assembly of CetZ1 and CetZ2 cytoskeletal structures is interlinked. Consistent with this, we also observed a significant dependency of CetZ2 cytoskeletal dynamics on the presence of CetZ1 in stationary phase. Together, the results suggest that the two proteins might directly or indirectly interact to counteract the CetZ1-based rod development pathway during stationary phase.

Interestingly, the effects of CetZ2 on preventing rod formation were highly specific to stationary phase. Overproduction of CetZ2 was only capable of inhibiting rod shape in stationary phase and not mid-log phase. Similarly, CetZ1 cytoskeletal structures were only affected by CetZ2 overproduction in stationary phase. We therefore believe it is likely that there are other factors or proteins specifically present in stationary phase that are necessary for CetZ2-based maintenance of plate cell shape (Fig. 7, right). The identity of these potential protein partners is currently unknown, but the involvement of other proteins would be unsurprising, given tubulin and FtsZ both recruit and work in conjunction with a myriad of other proteins (10, 29–32).

The other four *H*. *volcanii* CetZs (CetZ3-6) might represent candidates. However, previous proteomic studies did not detect upregulation of these in stationary phase (24). Although this does not rule out potential roles for them with CetZ2 in plate cell maintenance, it suggests that they are not likely to be the stationary-phase-specific factors implicated by our findings. CetZ5 has been hypothesized to be involved in the rod-to-plate transition based on proteomics that compared rods from early log and plates from late log (27), but this potential role has not yet been experimentally tested and would nevertheless occur well in advance of the mid-stationary-phase functions characterized here. Other potential proteins which may contribute to the maintenance of cell morphology during stationary phase include VolA and DdfA (27), both of which have been shown to be necessary for the rod-to-plate transition. Further genetic analyses or protein association and identification studies are hence warranted to begin characterizing the detailed mechanisms underpinning cell shape control in stationary phase.

In Haloarchaea, CetZ1 and CetZ2 homologs group into distinct protein families amongst multiple additional CetZ homologs in some species that are not so clearly grouped (17). Interestingly, CetZ2 was only identified in haloarchaea that also had CetZ1 (17). This raised the hypothesis that there is a functional dependency of CetZ2 on CetZ1, as identified here. Since most haloarchaea with both CetZ1 and CetZ2 are pleomorphic and rod forming, while those that had CetZ1 but not CetZ2 were not pleomorphic (17), the mechanism of coordination of shapeshifting through CetZ1 and CetZ2 is likely to be conserved across other Haloarchaea.

Our findings demonstrate that CetZ paralogs in haloarchaea can possess distinct and opposing functions, consistent with the phylogenetic separation and conservation of their two CetZ paralogous groups (1, 16, 17). The diversification of CetZ1 and CetZ2 function and their coordination is reminiscent of the specialized and coordinated functions of FtsZ1 and FtsZ2 in *H. volcanii* cell division (33) and of the multiplication, diversification, and specialization of tubulins in eukaryotes (34). Functional antagonism is rare among cytoskeletal proteins, but notably is a function of the vertebrate beta5-tubulin, which destabilizes microtubules when upregulated (35). We speculate that the specialized and opposing functions of archaeal CetZ1 and CetZ2 might resemble an early shift toward the evolution of obligate hetero-polymerization, such as seen between the active and inactive alpha- and beta-tubulins in microtubules of modern-day eukaryotes. This, coupled with the different and coordinated functions of FtsZ1 and FtsZ2 in *H. volcanii*, points toward a broader phenomenon of duplication and functional specialization of tubulin superfamily proteins in archaea.

## MATERIALS AND METHODS

### Materials and reagents

Unless otherwise specified, all chemicals were obtained from Sigma, reagents for cloning procedures were obtained from New England Biolabs and used according to the manufacturer’s instructions, and custom oligonucleotides were obtained from Integrated DNA Technologies. The Meridian Bioscience ISOLATE II PCR and Gel Kit and ISOLATE II Plasmid Mini Kit were used for purification of DNA from PCR reactions and agarose gels, and for plasmid extractions, respectively.

### *H. volcanii* strains and growth

A list of *H. volcanii* strains used in this study is available in Table S1. Cultures were prepared where indicated in standard culture media, either Hv-Cab (1), Hv-YPCab (18), or Hv-MinTE (36), which include trace elements, and incubated at 42°C with rotary shaking (200 rpm). If necessary, medium was supplemented with uracil (50 µg/mL) to fulfill auxotrophic requirements of Δ*pyrE2* strains, or with ʟ-Trp to induce expression of genes under the control of the p.*tna* promoter. For experiments that compared mid-log and stationary phase, cells were cultured in 10 mL Hv-YPCab medium by dilution of a steady mid-log starter culture to a theoretical starting OD_600_ of 0.0005. Previous work on the *cetZ1* and *cetZ2* deletion and overexpression strains (21), and the preliminary culturing of all strains in the current study showed that their growth rates were indistinguishable, so we sampled mid-log phase after a further 24 h of culturing, and then at 72 h, 96 h, and 120 h for early, mid, and late stationary phase, respectively (see Fig. 1).

### Genetic modification

The wild-type H26 (ID621) genetic background of *H. volcanii*, which is auxotrophic for uracil (Δ*pyrE2*), was used throughout this study. To generate the *cetZ1* and *cetZ2* knockout strains, pTA131-based plasmids for deletion of *cetZ1* (18) and *cetZ2* (1), containing the upstream and downstream flanks of *cetZ1* and *cetZ2* and the *pyrE2* gene, were used to transform H26 with selection on Hv-Cab agar. Transformation was done using the previously described method (37), and two-step (“pop-in-pop-out”) homologous recombination (38) was used for selection of transformants that showed complete deletion of the target gene.

To generate strains carrying chromosomal fusions of *cetZ2* with mTurquoise2, mChartreuse, and mLemon, the plasmids pTA131-*cetZ2mTq2*, pTA131-*cetZ2mChart,* and pTA131-*cetZ2mLem*, respectively, were synthesized (Genscript). The plasmids contained a 350 bp fragment encoding the C-terminal portion of *cetZ2* fused in frame to an open reading frame (ORF) containing the EG linker (semi-rigid) and the fluorescent protein (20), followed by the 350 bp flank immediately downstream of the *cetZ2* stop codon. These plasmids were used to transform H26 by the two-step method (38) to produce a scarless insertion of the EG linker and fluorescent proteins before the native *cetZ2* stop codon. The resulting chromosomal tag strains produced were *cetZ2-mTq2CHR*, *cetZ2-mChartCHR*, and *cetZ2-mLemCHR*.

### Construction of plasmids for gene expression in *H. volcanii*

A list of plasmids used in this study is available in Table S2, and relevant oligonucleotides are listed in Table S3. To generate plasmids for single and dual expression of CetZ fusion proteins, we used a recently described plasmid-based expression system (20). Briefly, the ORFs for *cetZ2* or *cetZ2*.E212A, excluding their stop codons, were PCR amplified using primers to incorporate NdeI and BamHI sites at the 5′ and 3′ ends, respectively, and the resulting PCR product and the vector backbone were ligated using T4 DNA ligase. The vector backbone was pHVID21 for C-terminal mTurquoise2 fusions with the EG linker, or pHVID20 for C-terminal YPet fusions with the EG linker.

To generate plasmids for dual expression of two tagged proteins, pHJB5 and pHJB11 (containing *cetZ2*-YPet and *cetZ2*.E212A-YPet, respectively) were used as vector backbones for insertion of *cetZ1*-mTq2 and *cetZ1*.E218A-mTq2 ORFs positioned immediately downstream of the *cetZ2* ORFs as described (20). The ORFs for *cetZ2-*YPet, *cetZ2*.E212A-YPet, *cetZ1*-mTq2, and *cetZ1*.E218A*-*mTq2 were sourced from pHJB5, pHJB11, pHVID135, and pHJB13, respectively.

Plasmids for dual expression of one untagged CetZ and one tagged CetZ were prepared using plasmids for expression of *cetZ2*-mTq2, *cetZ2*, or *cetZ2*.E212A (pHJB6, pTA962-*cetZ2*, and pTA962-*cetZ2*.E212A, respectively) as backbones. pHJB6 was subjected to restriction digest by NheI and NotI, while pTA962-*cetZ2* and pTA962-*cetZ2*.E212A were digested using BamHI and NotI. ORFs for *cetZ1* and *cetZ1*.E218A were amplified by PCR incorporating an NheI site at the 5′ end, and the ORF for *cetZ1*-mTq2 was amplified to incorporate a BglII site at the 5′ end. After restriction digest with the relevant enzymes, the various *cetZ1* ORFs were ligated as described above into the backbones, generating plasmids pHJB43-46 for dual expression of *cetZ2/cetZ1-*mTq2, *cetZ2*.E212A/*cetZ1-*mTq2, *cetZ2-*mTq2/*cetZ1*, and *cetZ2-*mTq2/*cetZ1*.E218A, respectively. All plasmid regions originating from PCR products were sequence verified (Australian Genome Research Facility). Plasmids were passaged through *Escherichia coli* strain C2925 for demethylation before transformation of *H. volcanii*.

### Soft-agar motility assay

*H. volcanii* was maintained in exponential growth in liquid culture (Hv-Cab medium) for at least 2 days. Following this, cultures were normalized to OD_600_ ~ 0.1, and 2 µL of cells were spotted onto the surface of Hv-Cab agar (0.2% [wt/vol]). Soft-agar plates were incubated statically at 42°C for 4 days before extracting cells from the halo edge or inner halo (approximately 5 mm from the inoculation site) as described (21) for further analysis by microscopy.

### Microscopy

For all microscopy, 2 µL of cells was spotted onto a 1.5% (wt/vol) agarose pad containing 18% buffered salt water (38), and a glass coverslip (#1.5) was placed on top before imaging. Unless otherwise noted in the figure legends, phase contrast and fluorescent images were acquired using a V3 DeltaVision Elite inverted fluorescence microscope (GE Healthcare), with CFP (excitation filter = 400–453 nm; emission filter = 463–487 nm) and YFP (excitation filter = 497–527 nm; emission filter = 537–549 nm) filter sets. Images were acquired using a 100 × 1.4 NA Plan Apo objective. Time-lapse images were recorded over 30 min with 1 min intervals, using an incubated microscope stage at 37°C. Exposure and acquisition settings were maintained between strains and replicates within each experiment. Alternatively, a Nikon (Ti2-E) N-STORM v5, equipped with a 100 × 1.49 NA oil objective, was used to capture brightfield and fluorescence images.

3D-SIM was carried out as previously described (22, 23), using a DeltaVision OMX SR (GE Healthcare) microscope with a 60× Plan 1.42 NA objective, a PCO sCMOS camera, 125 nm Z-step size, with a DAPI excitation filter (405 nm) and AF488 emission filter (504–552 nm). Raw images were reconstructed using SoftWorX (Applied Precision, GE), and then maximum intensity projections were generated in FIJI (39), and 3D renders were generated using Imaris (V 7.6.4 BitPlane Scientific). Small example fields of view were then cropped to display the main protein localization characteristics of the sample, and the whole data set was inspected and subjected to quantitative image analysis.

### Image analysis

All shape analysis of cells was conducted using phase-contrast images analyzed using MicrobeJ v5.13m (40) in FIJI (39). To detect cell outlines using phase-contrast images, default settings were applied while restricting area between 0.8 max µm^2^ and sinuosity between 0 and 2. For all analyses of fluorescence, raw images and time-lapse data were first processed by applying the Background Subtraction function (rolling ball size = 8 pixels). Time-lapse data were further processed using Bleach Correction (algorithm = Histogram Matching) and StackReg (transformation = Translation).

For static localization analysis, the MicrobeJ “foci” detection method with default settings was used with tolerance set to 1,000, *Z*-score was set to 25, and area was restricted between 0 and 0.5 µm^2^. Mean whole-cell fluorescence intensity (Bacteria.Intensity.ch2.mean), the number of localizations per cell (Bacteria.Maxima), and localization area (Maxima.Shape.area), maximum localization intensity (Maxima.Intensity.max: the highest pixel value within the area of an identified focus), and mean localization intensity (Maxima.Intensity.mean: the sum of pixel values normalized by the number of pixels within the area of the focus) were extracted from MicrobeJ result tables. Localization heat maps were generated using the XYCellDensity function.

For tracking of localizations, TrackMate2 v7.7.2 (41, 42) was used. This tracks the movement of localizations in the *XY* plane only, excluding the *Z* plane. The LoG detector was used to detect foci, using an estimated focus diameter of 0.5 µm and a quality threshold of 100. For tracking, the LAP tracker was used, allowing for frame-to-frame linking, track segment gap closing, track segment splitting, and track segment merging, with a maximum distance of 0.5 µm and a maximum frame gap of 2. Mean localization speed of individual trajectories was extracted from the TrackMate2 results interface, and trajectory patterns were manually scored.

For colocalization analysis, EzColocalization (43) was used. Manual adjustment of thresholds using the default algorithm was required for accurate detection of fluorescent channels, ensuring that weak or background fluorescence was not included in the thresholding. Auto thresholding with the default algorithm and watershed segmentation was used for cell identification using the phase-contrast channel. Fluorescent channels were aligned, and the background was subtracted using a rolling-ball size of 8. All remaining settings and parameters were used as default.

### SDS-PAGE and western blotting

Samples were prepared for SDS-PAGE by resuspending whole cell pellets in lysis buffer (20 mM Tris-HCl, pH 7.5, 1× DNase I [ThermoFisher], 1× EDTA-free protease inhibitor [ThermoFisher]) and incubating for 30 min at room temperature. Following that, 4× SDS-PAGE sample buffer (250 mM Tris-HCl, pH 6.8, 40% [vol/vol] glycerol, 4% [wt/vol] SDS, 0.04% [wt/vol] Bromophenol blue, and 20% [vol/vol] 2-mercaptoethanol) was added to a final concentration of 1×, using a volume that would give a theoretical OD_600_ of 5 based on the original culture. The samples were heated at 95°C for 5 min then vortexed for 1 min.

CetZ1-6×His and CetZ2-6×His proteins (1) were purified and quantified by *A*_280_ in a previous study (1) and diluted in 1× SDS-PAGE sample buffer to 200 ng/μL and 50 ng/μL, respectively. Following this, 1:2 serial dilutions in 1× SDS-PAGE sample buffer were carried out for each protein. Samples were loaded onto a 4%–20% Mini-PROTEAN TGX pre-cast polyacrylamide gel (Bio-Rad), using the Broad Range Blue Pre-stained Protein Standard (Νew England Biolabs). Electrophoresis was carried out at 100 V for 85 min with a Bio-Rad Mini-Protean3 electrophoresis system.

For western blotting, proteins separated by SDS-PAGE were transferred to a 0.2 µm nitrocellulose membrane using the Trans-Blot Turbo mini transfer system (Bio-Rad). The membrane was stained with Ponceau S (0.1% [wt/vol] Ponceau S in 5% [vol/vol] acetic acid) for 5 min, and the excess stain was removed by rinsing with Milli-Q water to obtain a total protein stain. Following this, the nitrocellulose membrane was blocked in 5% (wt/vol) skim milk powder in TBST (50 mM Tris-HCl, pH 7.4, 150 mM NaCl, and 0.05% [vol/vol] Tween-20) for 2 h at room temperature. Primary antibodies (1) against CetZ1 or CetZ2 were diluted 1/500 in TBST for incubation with the membrane overnight at 4°C. The membrane was washed three times in TBST and incubated with the secondary antibody (donkey anti-rabbit IgG HRP conjugate, AbCam 16284) diluted 1/5,000 in TBST for 2 h at room temperature. The membrane was washed five times in TBST and incubated with the SuperSignal West Pico PLUS chemiluminescent substrate (ThermoFisher) for 5 min before imaging of the membrane using an Amersham Imager 600 (GE Healthcare).

To calculate the number of molecules of CetZ1/2 per cell, six replicate cultures were grown in Hv-YPCab medium and sampled after 24 and 96 h. Band intensity from western blotting analysis (Fig. 1D and E) was determined by measuring the raw integrated density in ImageJ (39) and subtracting the raw integrated density of the background. The same measurements were conducted for pure protein standards of known concentrations. Band intensities of the standards were used to determine the amount of protein per OD unit of cells in mid-log (24 h) and stationary phase (96 h) samples. The number of cells per OD unit in mid-log and stationary phase was calculated using serial dilutions and counting the number of colony-forming units from the same six culture replicates used for western blotting samples. Finally, this was used to determine the number of CetZ1 and CetZ2 molecules per cell.

## SUPPLEMENTAL MATERIAL

Supplemental Material mbio.00854-26-s0001.pdf Supplemental figures, tables, and video legends.

Video S1 mbio.00854-26-s0002.mp4 3D render of CetZ2-mTq2 in an *H. volcanii* cell.

Video S2 mbio.00854-26-s0003.avi Time-lapse imaging of CetZ2-mTq2 localization dynamics in early, mid, and late stationary phase cells.

Video S3 mbio.00854-26-s0004.avi Tracking of CetZ2-mTq2 localizations in *H. volcanii* stationary phase cells.

Video S4 mbio.00854-26-s0005.avi Time-lapse imaging of tagged CetZ2 produced from its native chromosomal locus in stationary phase cells.

Video S5 mbio.00854-26-s0006.avi Time-lapse imaging of CetZ2-mTq2 and CetZ2-E212A-mTq2 in the presence or absence of CetZ1 in stationary phase cells.

Video S6 mbio.00854-26-s0007.avi Time-lapse imaging of the GTPase mutant CetZ2.E212A-mTq2 in stationary phase cells.

Video S7 mbio.00854-26-s0008.avi Time-lapse imaging of CetZ1-mTq2 and CetZ1-E218A-mTq2 in stationary phase cells in the presence or absence of CetZ2.

Video S8 mbio.00854-26-s0009.avi Time-lapse imaging of CetZ1-mTq2 or CetZ2-mTq2 in stationary phase cells co-producing the alternate CetZ or GTPase mutant.

ASM does not own the copyrights to Supplemental Material that may be linked to, or accessed through, an article. The authors have granted ASM a non-exclusive, world-wide license to publish the Supplemental Material files. Please contact the corresponding author directly for reuse.

## References

[1] Duggin IG, Aylett CHS, Walsh JC, Michie KA, Wang Q, Turnbull L, Dawson EM, Harry EJ, Whitchurch CB, Amos LA, Löwe J. 2015. CetZ tubulin-like proteins control archaeal cell shape. Nature 519:362–365. doi:10.1038/nature13983 25533961 PMC4369195

[2] Liao Y, Ithurbide S, de Silva RT, Erdmann S, Duggin IG. 2018. Archaeal cell biology: diverse functions of tubulin-like cytoskeletal proteins at the cell envelope. Emerg Top Life Sci 2:547–559. doi:10.1042/ETLS20180026 33525829

[3] Nogales E, Downing KH, Amos LA, Lowe J. 1998. Tubulin and FtsZ form a distinct family of GTPases. Nat Struct Mol Biol 5:451–458. doi:10.1038/nsb0698-451 9628483

[4] Desai A, Mitchison TJ. 1997. Microtubule polymerization dynamics. Annu Rev Cell Dev Biol 13:83–117. doi:10.1146/annurev.cellbio.13.1.83 9442869

[5] Mitchison TJ. 1993. Localization of an exchangeable GTP binding site at the plus end of microtubules. Science 261:1044–1047. doi:10.1126/science.8102497 8102497

[6] Mitchison T, Kirschner M. 1984. Dynamic instability of microtubule growth. Nature 312:237–242. doi:10.1038/312237a0 6504138

[7] Nogales E. 2001. Structural insight into microtubule function. Annu Rev Biophys Biomol Struct 30:397–420. doi:10.1146/annurev.biophys.30.1.397 11441808

[8] Mukherjee A, Lutkenhaus J. 1994. Guanine nucleotide-dependent assembly of FtsZ into filaments. J Bacteriol 176:2754–2758. doi:10.1128/jb.176.9.2754-2758.1994 8169229 PMC205420

[9] Mukherjee A, Lutkenhaus J. 1998. Dynamic assembly of FtsZ regulated by GTP hydrolysis. EMBO J 17:462–469. doi:10.1093/emboj/17.2.462 9430638 PMC1170397

[10] Adams DW, Errington J. 2009. Bacterial cell division: assembly, maintenance and disassembly of the z ring. Nat Rev Microbiol 7:642–653. doi:10.1038/nrmicro2198 19680248

[11] Barton NR, Goldstein LS. 1996. Going mobile: microtubule motors and chromosome segregation. Proc Natl Acad Sci USA 93:1735–1742. doi:10.1073/pnas.93.5.1735 8700828 PMC39850

[12] Appert-Rolland C, Ebbinghaus M, Santen L. 2015. Intracellular transport driven by cytoskeletal motors: general mechanisms and defects. Phys Rep 593:1–59. doi:10.1016/j.physrep.2015.07.001

[13] Hirokawa N, Noda Y, Tanaka Y, Niwa S. 2009. Kinesin superfamily motor proteins and intracellular transport. Nat Rev Mol Cell Biol 10:682–696. doi:10.1038/nrm2774 19773780

[14] Garcin C, Straube A. 2019. Microtubules in cell migration. Essays Biochem 63:509–520. doi:10.1042/EBC20190016 31358621 PMC6823166

[15] Kaverina I, Straube A. 2011. Regulation of cell migration by dynamic microtubules. Semin Cell Dev Biol 22:968–974. doi:10.1016/j.semcdb.2011.09.017 22001384 PMC3256984

[16] Aylett CH, Duggin IG. 2017. The tubulin superfamily in archaea, p 393–417. In Löwe J, Amos L (ed), Prokaryotic cytoskeletons. Springer, Cham. 10.1007/978-3-319-53047-5_14. 28500534

[17] Brown HJ, Duggin IG. 2023. Diversity and potential multifunctionality of archaeal CetZ tubulin-like cytoskeletal proteins. Biomolecules 13:134. doi:10.3390/biom13010134 36671519 PMC9856176

[18] de Silva RT, Abdul-Halim MF, Pittrich DA, Brown HJ, Pohlschroder M, Duggin IG. 2021. Improved growth and morphological plasticity of *Haloferax volcanii.* Microbiology 167:001012. doi:10.1099/mic.0.001012 33459585 PMC8131023

[19] Li Z, Kinosita Y, Rodriguez-Franco M, Nußbaum P, Braun F, Delpech F, Quax TEF, Albers S-V. 2019. Positioning of the motility machinery in halophilic archaea. mBio 10:e00377-19. doi:10.1128/mBio.00377-19 31064826 PMC6509185

[20] Ithurbide S, de Silva RT, Brown HJ, Shinde V, Duggin IG. 2024. A vector system for single and tandem expression of cloned genes and multi-colour fluorescent tagging in *Haloferax volcanii.* Microbiology 170:001461. doi:10.1099/mic.0.001461 38787390 PMC11165654

[21] Brown HJ, Islam MI, Ruan J, Baker MA, Ithurbide S, Duggin IG. 2024. CetZ1-dependent assembly and positioning of the motility machinery in haloarchaea. bioRxiv. doi:10.1101/2024.05.02.592137 PMC13644373 42611957

[22] Brown HJ, Duggin IG. 2024. MinD proteins regulate CetZ1 localization in *Haloferax volcanii*. Front Microbiol, 15:1474697. doi:10.3389/fmicb.2024.1474697 39651350 PMC11621097

[23] de Silva RT, Shinde V, Brown HJ, Liao Y, Duggin IG. 2024. Dynamic self-association of archaeal tubulin-like protein CetZ1 drives *Haloferax volcanii* morphogenesis. bioRxiv. doi:10.1101/2024.04.08.588506

[24] Cerletti M, Giménez MI, Tröetschel C, D’ Alessandro C, Poetsch A, De Castro RE, Paggi RA. 2018. Proteomic study of the exponential-stationary growth phase transition in the haloarchaea natrialba magadii and *Haloferax volcanii*. Proteomics 18:e1800116. doi:10.1002/pmic.201800116 29888524

[25] Large A, Stamme C, Lange C, Duan Z, Allers T, Soppa J, Lund PA. 2007. Characterization of a tightly controlled promoter of the halophilic archaeon *Haloferax volcanii* and its use in the analysis of the essential cct1 gene. Mol Microbiol 66:1092–1106. doi:10.1111/j.1365-2958.2007.05980.x 17973910

[26] Curtis Z, Escudeiro P, Mallon J, Leland O, Rados T, Dodge A, Andre K, Kwak J, Yun K, Isaac B, Martinez Pastor M, Schmid AK, Pohlschroder M, Alva V, Bisson A. 2024. Halofilins as emerging bactofilin families of archaeal cell shape plasticity orchestrators. Proc Natl Acad Sci USA 121:e2401583121. doi:10.1073/pnas.2401583121 39320913 PMC11459167

[27] Schiller H, Hong Y, Kouassi J, Rados T, Kwak J, DiLucido A, Safer D, Marchfelder A, Pfeiffer F, Bisson A, Schulze S, Pohlschroder M. 2024. Identification of structural and regulatory cell-shape determinants in *Haloferax volcanii*. Nat Commun 15:1414. doi:10.1038/s41467-024-45196-0 38360755 PMC10869688

[28] Löwe J, Amos LA. 2009. Evolution of cytomotive filaments: the cytoskeleton from prokaryotes to eukaryotes. Int J Biochem Cell Biol 41:323–329. doi:10.1016/j.biocel.2008.08.010 18768164

[29] Osawa M, Erickson HP. 2013. Liposome division by a simple bacterial division machinery. Proc Natl Acad Sci USA 110:11000–11004. doi:10.1073/pnas.1222254110 23776220 PMC3703997

[30] Szwedziak P, Wang Q, Bharat TAM, Tsim M, Löwe J. 2014. Architecture of the ring formed by the tubulin homologue FtsZ in bacterial cell division. eLife 3:e04601. doi:10.7554/eLife.04601 25490152 PMC4383033

[31] Yang X, Lyu Z, Miguel A, McQuillen R, Huang KC, Xiao J. 2017. GTPase activity-coupled treadmilling of the bacterial tubulin FtsZ organizes septal cell wall synthesis. Science 355:744–747. doi:10.1126/science.aak9995 28209899 PMC5851775

[32] Akhmanova A, Kapitein LC. 2022. Mechanisms of microtubule organization in differentiated animal cells. Nat Rev Mol Cell Biol 23:541–558. doi:10.1038/s41580-022-00473-y 35383336

[33] Liao Y, Ithurbide S, Evenhuis C, Löwe J, Duggin IG. 2021. Cell division in the archaeon *Haloferax volcanii* relies on two FtsZ proteins with distinct functions in division ring assembly and constriction. Nat Microbiol 6:594–605. doi:10.1038/s41564-021-00894-z 33903747 PMC7611241

[34] Brown HJ, Shinde VD, Bosi L, Duggin IG. 2025. Evolution of the cytoskeleton: emerging clues from the diversification and specialisation of archaeal cytoskeletal proteins. Curr Opin Cell Biol 95:102557. doi:10.1016/j.ceb.2025.102557 40513207

[35] Bhattacharya R, Cabral F. 2004. A ubiquitous beta-tubulin disrupts microtubule assembly and inhibits cell proliferation. Mol Biol Cell 15:3123–3131. doi:10.1091/mbc.e04-01-0060 15121885 PMC452570

[36] Liao Y, Vogel V, Hauber S, Bartel J, Alkhnbashi OS, Maaß S, Schwarz TS, Backofen R, Becher D, Duggin IG, Marchfelder A. 2021. CdrS is a global transcriptional regulator influencing cell division in *Haloferax volcanii*. bioRxiv. doi:10.1101/2021.05.11.443588 PMC8406309 34253062

[37] Cline SW, Lam WL, Charlebois RL, Schalkwyk LC, Doolittle WF. 1989. Transformation methods for halophilic archaebacteria. Can J Microbiol 35:148–152. doi:10.1139/m89-022 2497937

[38] Allers T, Ngo H-P, Mevarech M, Lloyd RG. 2004. Development of additional selectable markers for the halophilic archaeon *Haloferax volcanii* based on the leuB and trpA genes. Appl Environ Microbiol 70:943–953. doi:10.1128/AEM.70.2.943-953.2004 14766575 PMC348920

[39] Schindelin J, Arganda-Carreras I, Frise E, Kaynig V, Longair M, Pietzsch T, Preibisch S, Rueden C, Saalfeld S, Schmid B, Tinevez J-Y, White DJ, Hartenstein V, Eliceiri K, Tomancak P, Cardona A. 2012. Fiji: an open-source platform for biological-image analysis. Nat Methods 9:676–682. doi:10.1038/nmeth.2019 22743772 PMC3855844

[40] Ducret A, Quardokus EM, Brun YV. 2016. MicrobeJ, a tool for high throughput bacterial cell detection and quantitative analysis. Nat Microbiol 1:16077. doi:10.1038/nmicrobiol.2016.77 27572972 PMC5010025

[41] Ershov D, Phan M-S, Pylvänäinen JW, Rigaud SU, Le Blanc L, Charles-Orszag A, Conway JRW, Laine RF, Roy NH, Bonazzi D, Duménil G, Jacquemet G, Tinevez J-Y. 2022. TrackMate 7: integrating state-of-the-art segmentation algorithms into tracking pipelines. Nat Methods 19:829–832. doi:10.1038/s41592-022-01507-1 35654950

[42] Tinevez J-Y, Perry N, Schindelin J, Hoopes GM, Reynolds GD, Laplantine E, Bednarek SY, Shorte SL, Eliceiri KW. 2017. TrackMate: an open and extensible platform for single-particle tracking. Methods 115:80–90. doi:10.1016/j.ymeth.2016.09.016 27713081

[43] Stauffer W, Sheng H, Lim HN. 2018. EzColocalization: an ImageJ plugin for visualizing and measuring colocalization in cells and organisms. Sci Rep 8:15764. doi:10.1038/s41598-018-33592-8 30361629 PMC6202351

