# Supplementary material for: Archaeal tubulin-like proteins CetZ1 and CetZ2 have opposing effects on cell morphology during the growth cycle of Haloferax volcanii
Source: mBio. 2026 Aug 7;17(9):e00854-26. doi: 10.1128/mbio.00854-26 (PMC13556191; doi:10.1128/mbio.00854-26)
Supplement: Supplementary file 1 [file mbio.00854-26-s0001.pdf]

Supplementary Material

Archaeal tubulin-like proteins CetZ1 and CetZ2 have opposing effects on cell morphology during the growth cycle of *Haloferax volcanii*

Hannah J. Brown[1] & Iain G. Duggin[1]

[1]The Australian Institute for Microbiology and Infection, University of Technology Sydney, NSW, 2007, Australia.

Correspondance: Hannah.Brown@uts.edu.au, Iain.Duggin@uts.edu.au

CONTENTS:

Supplementary Figures:

1. CetZ1 and CetZ2 cellular concentrations are not dependent on one another.
2. Function and expression of cetZ2-mTq2 from the chromosome during stationary phase transition and motility assays.
3. Analysis of plasmid-based expression of cetZ2 or cetZ2 fusion proteins under various experimental conditions.
4. Localisation and dynamics of plasmid produced CetZ2-mTq2 using varying concentrations of L-Tryptophan for induction.
5. Chromosomal tagging of CetZ2 with mChartreuse and mLemon.
6. Extended analysis of CetZ1, CetZ2, CetZ1.E218A, and CetZ2.E212A localisation during mid-stationary phase
7. A mutation in the CetZ2 GTPase active site alters subcellular localization and blocks dynamic movement in stationary phase.
8. Localisation of CetZ1, CetZ2, CetZ1.E218A, and CetZ2.E212A during mid-log growth.

Supplementary Tables:

1. Strains used in this study
2. Plasmids used in this study
3. Oligonucleotides used in this study

Legends for Supplementary Videos

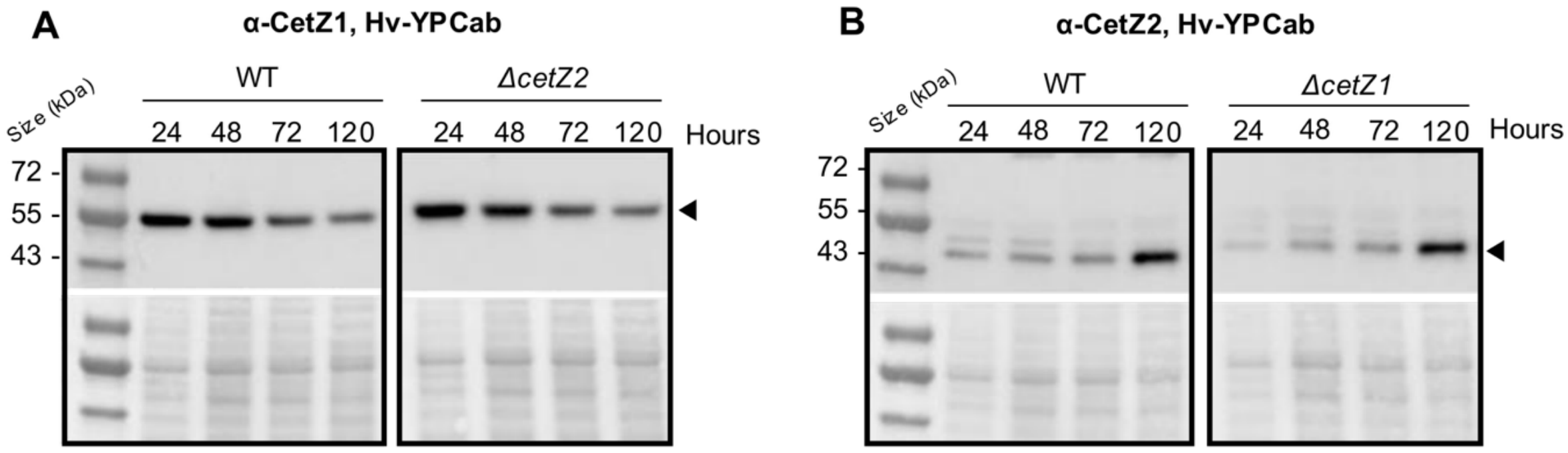

Figure S1. CetZ1 and CetZ2 cellular concentrations are not dependent on one another. A) Cultures of wild-type and the *cetZ2* deletion strain containing pTA962 were grown in Hv-YPCab medium and whole cell lysate samples were taken after 24, 48, 72, and 120 hours of culturing, and biomass-normalised ($OD_{600\ nm}$) for western blotting analysis using an antibody against CetZ1. The arrowhead indicates band for CetZ1, and bottom panel shows Ponceau S staining used to confirm equal loading of samples. **B)** As in A, but for analysis of CetZ2 expression in the wild-type and *cetZ1* deletion background. Solid arrowhead indicates band for CetZ2.

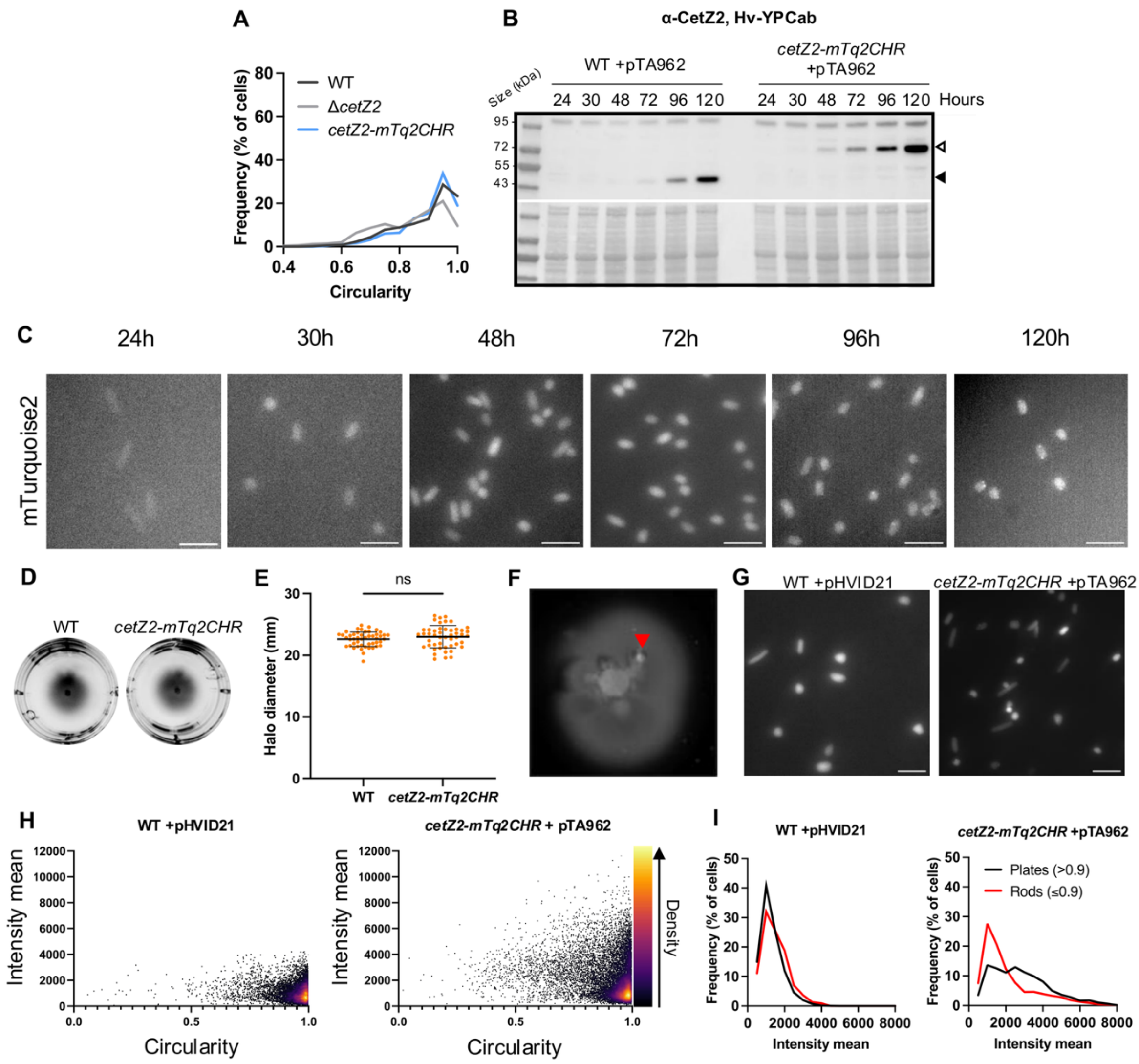

Figure S2. Function and expression of cetZ2-mTq2 from the chromosome during stationary phase transition and motility assays. A) Cell circularity measurements were obtained from phase-contrast imaging of *cetZ2-mTq2CHR* +pTA962 grown in Hv-YPCab medium supplemented with 0.2 mM ʟ-tryptophan and sampled in late stationary phase (120 h). **B)** WT +pTA962 and *cetZ2-mTq2CHR* +pTA962 were grown in Hv-YPCab and whole cell lysate samples were taken at 24, 30, 48, 72, 96, and 120 h of culturing for western blotting analysis using an antibody against CetZ2. Solid arrowhead indicates band for CetZ2, empty arrowhead indicates band for the CetZ2-mTq2 fusion protein. **C)** Representative fluorescence microscopy images of *cetZ2-mTq2CHR* + pTA962 grown in Hv-YPCab liquid medium throughout the growth cycle, used to generate graph in Fig. 2D. Exposure and brightness and contrast settings are maintained across timepoints. Scale bar=5 µm. **D)** WT +pTA962 and *cetZ2-mTq2CHR* +pTA962 were inoculated on Hv-Cab soft-agar (0.2%) and incubated for 3 days at 42℃. **E)** The diameter of motility halos was measured for eight culture replicates with 6 technical replicates each. Unpaired t-test was used for statistical analysis, ns=not significant. **F)** WT +pHVID21 (for expression of free mTq2) and *cetZ2-mTq2CHR* +pTA962 were inoculated on soft-agar containing 1 mM L-Tryptophan as in **D**, and were extracted from the inner halo region, approximately 5 mm from the inoculation site after four days. Red arrowhead indicates sample location at inner halo. **F)** Extracted cells were observed by fluorescence microscopy. Scale bar=5 µm. **H)** The average whole-cell fluorescence intensity and circularity (measured using phase-contrast images, not shown) of individual cells from **G** are displayed in XY scatter plots. Individual data points are coloured by density. **I)** Cells with a circularity >0.9 were categorised as plates, and those with circularity ≤0.9 were categorised as rods. The mean fluorescence intensity in plates and rods is represented as a distribution. Data in **H** and **I** was pooled from four biological replicates each with at least two technical replicates. WT + pHVID21: n=7468; *cetZ2mTq2CHR* + pTA962: n=13400.

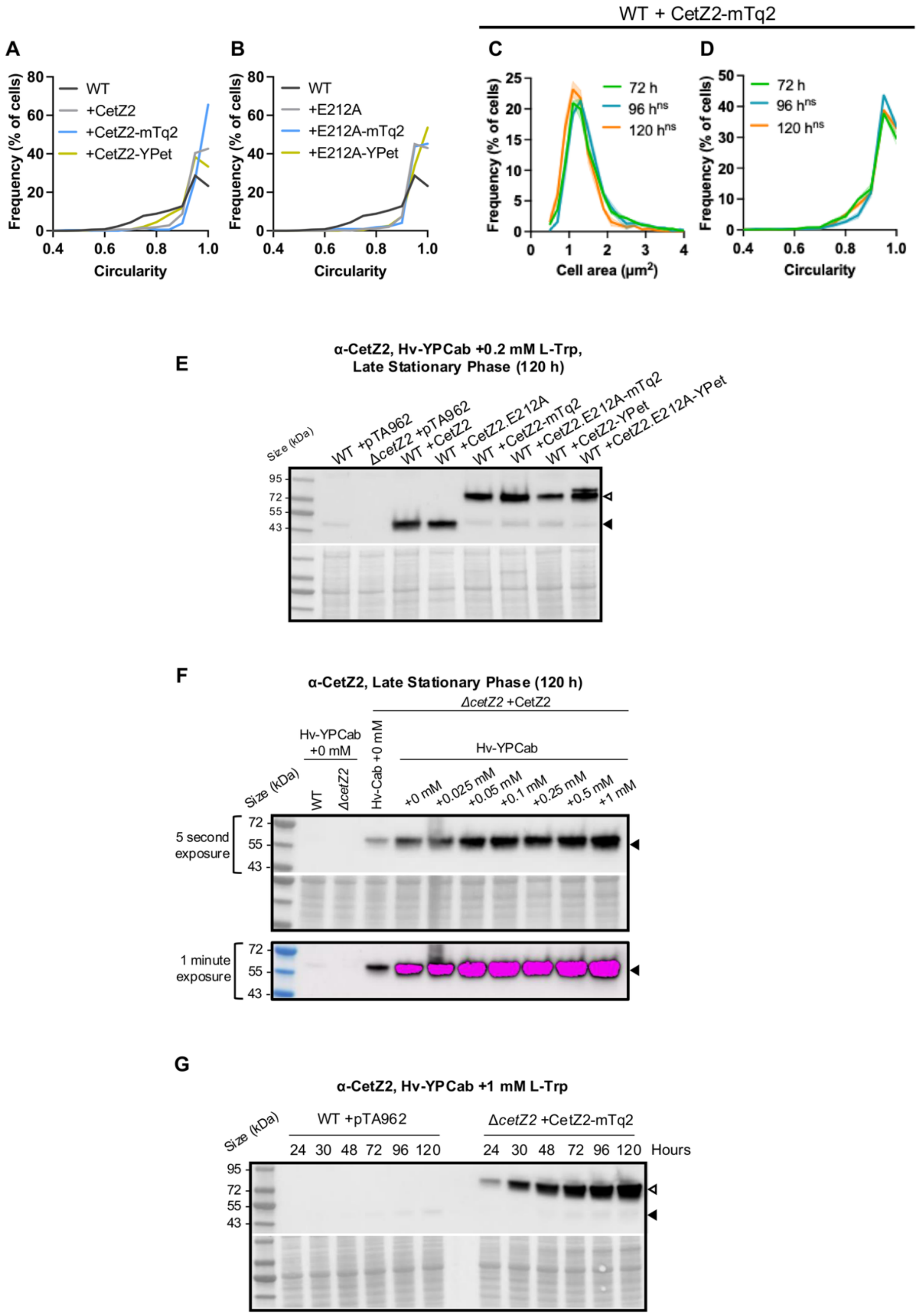

Figure S3. ***Analysis of plasmid-based expression of cetZ2 or cetZ2 fusion proteins under various experimental conditions.*** **A)** CetZ2 or CetZ2 fusion proteins were produced in Δ*cetZ2* using 0.2 mM L-tryptophan and were sampled during late stationary phase and for phase-contrast microscopy (not shown). The cell circularity for each strain was measured to determine CetZ2 fusion protein functionality in comparison to expression of untagged CetZ2 from the same vector backbone. **B)** As in **A,** but for CetZ2.E212A and CetZ2.E212A fusion proteins. **C)** Cell area and **D)** circularity analysis of wildtype cells expressing CetZ2-mTq2 in early, mid, and

late stationary phases. Bold lines indicate mean of independent replicates, shading indicates standard error. One-way ANOVA was used as a statistical test to compare the means of independent replicates from 96 h and 120 h to 72 h (ns=not significant). The number of replicates and cells is as in Figure 3b. **E)** The CetZ2 and CetZ2.E212A fusion proteins in **A** and **B** were produced in the wild-type (H26) background by induction with 0.2 mM ʟ-tryptophan, and whole cell lysates were taken during late stationary phase (120 h). These were analysed by western blotting to examine proteolytic cleavage. Since induction of CetZ2 or CetZ2 fusion protein production with 0.2 mM L-tryptophan resulted in strong overproduction in **E** and caused an overexpression-like phenotype (increased circularity) in **A**, we sought to compare varying levels of ʟ-tryptophan induction to the native level of CetZ2. **F)** The wild-type (H26 +pTA962) or Δ*cetZ2* (+pTA962) were grown in Hv-Cab or Hv-YPCab medium with the indicated concentrations of ʟ-tryptophan and whole cell lysates were sampled during late stationary phase for western blotting. Longer exposure showed a band for wild-type in Hv-YPCab +0 mM L-tryptophan (lane 2) which was not visible in the shorter exposure, however longer exposure resulted in saturation of chemiluminescent signal from other lanes (indicated in pink). **G)** Wild-type (H26 +pTA962) and Δ*cetZ2* producing CetZ2-mTq2 from pHJB6 were grown in Hv-YPCab supplemented with 1 mM L-Tryptophan and sampled for western blotting analysis at the indicated timepoints throughout the growth cycle. In **E**-**G**, all blots have used an antibody against CetZ2. Solid arrowheads indicate size for CetZ2, empty arrowheads indicate size for CetZ2 fusion proteins. Bottom panels show Ponceau S total protein staining.

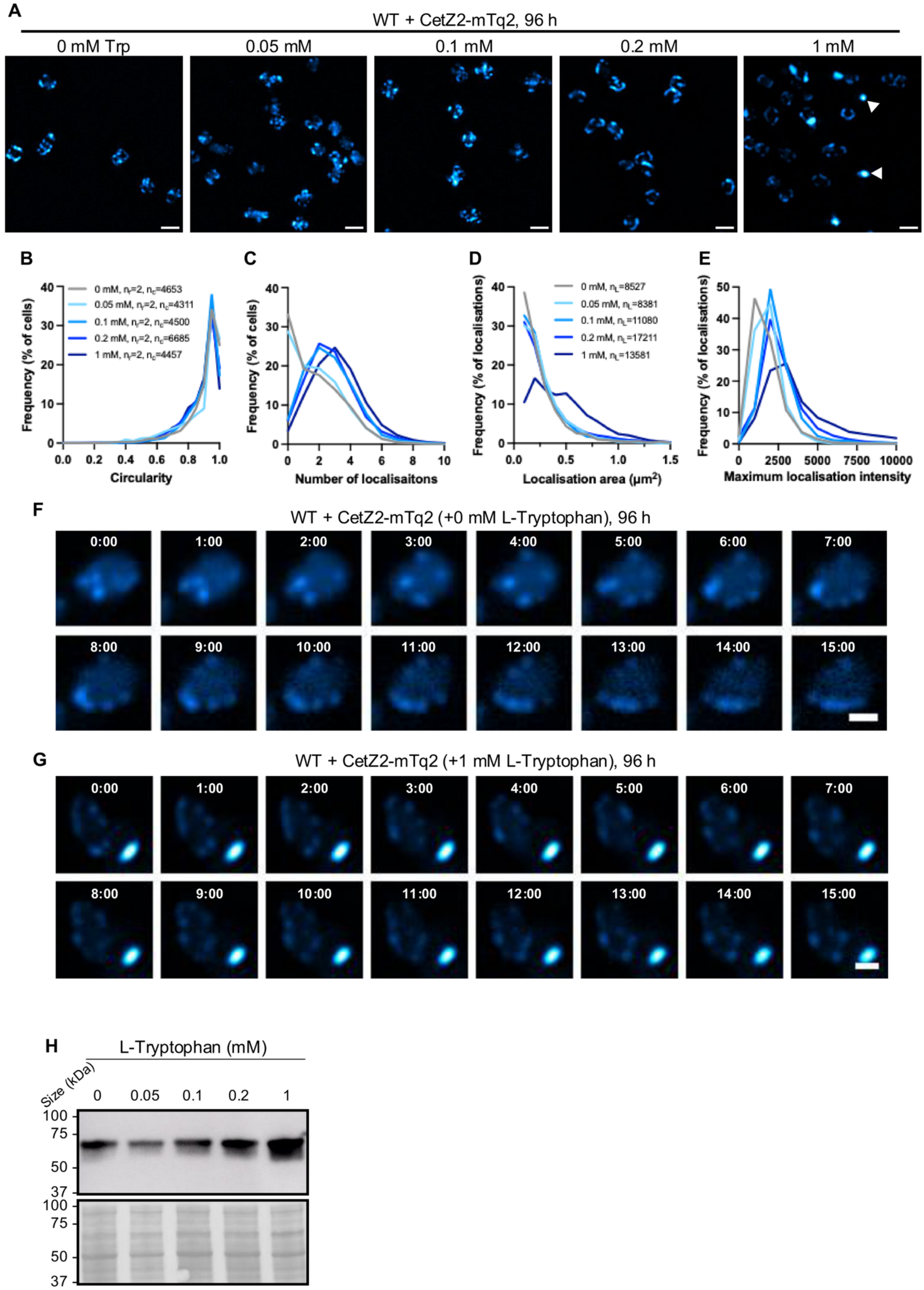

Figure S4. ***Localisation and dynamics of plasmid-produced CetZ2-mTq2 using varying concentrations of L-Trp for induction.*** **A)** WT + pHJB6 (for expression of CetZ2-mTq2) was grown in Hv-YPCab medium supplemented

with 0, 0.05, 0.1, 0.2, or 1 mM L-tryptophan, and imaged using fluorescence microscopy during mid-stationary phase (96 h). Scale bar = 2 µm. Images are brightness and contrast adjusted for viewability of CetZ2-mTq2 localization characteristics. **B)** Frequency distributions showing cell circularity, **C)** Number of localizations per cell, **D)** localization area, and **E)** mean localization intensity. **F)** Time-lapse imaging (1 min intervals) of plasmid expressed CetZ2-mTq2, in Hv-YPCab medium without added L-Trp. **G)** Time lapse with 1 mM L-Trp induction. The large aggregates which only occurred when at least 1 mM L-Trp was used for induction were static and are not likely to be representative of CetZ2 localisation or dynamics at native levels. The number of independent replicates (n_r) and number of pooled cells (n_c) are indicated in the key of panel **B** and the number of localisations from all pooled cells (n_L) is indicated in panel **D**. Values of n_r applies to panels **B-G**, n_c applies to **B** and **C**, and n_L applies to **D** and **E. F)** Western blotting analysis using an antibody against CetZ2 for cells shown in **A.**

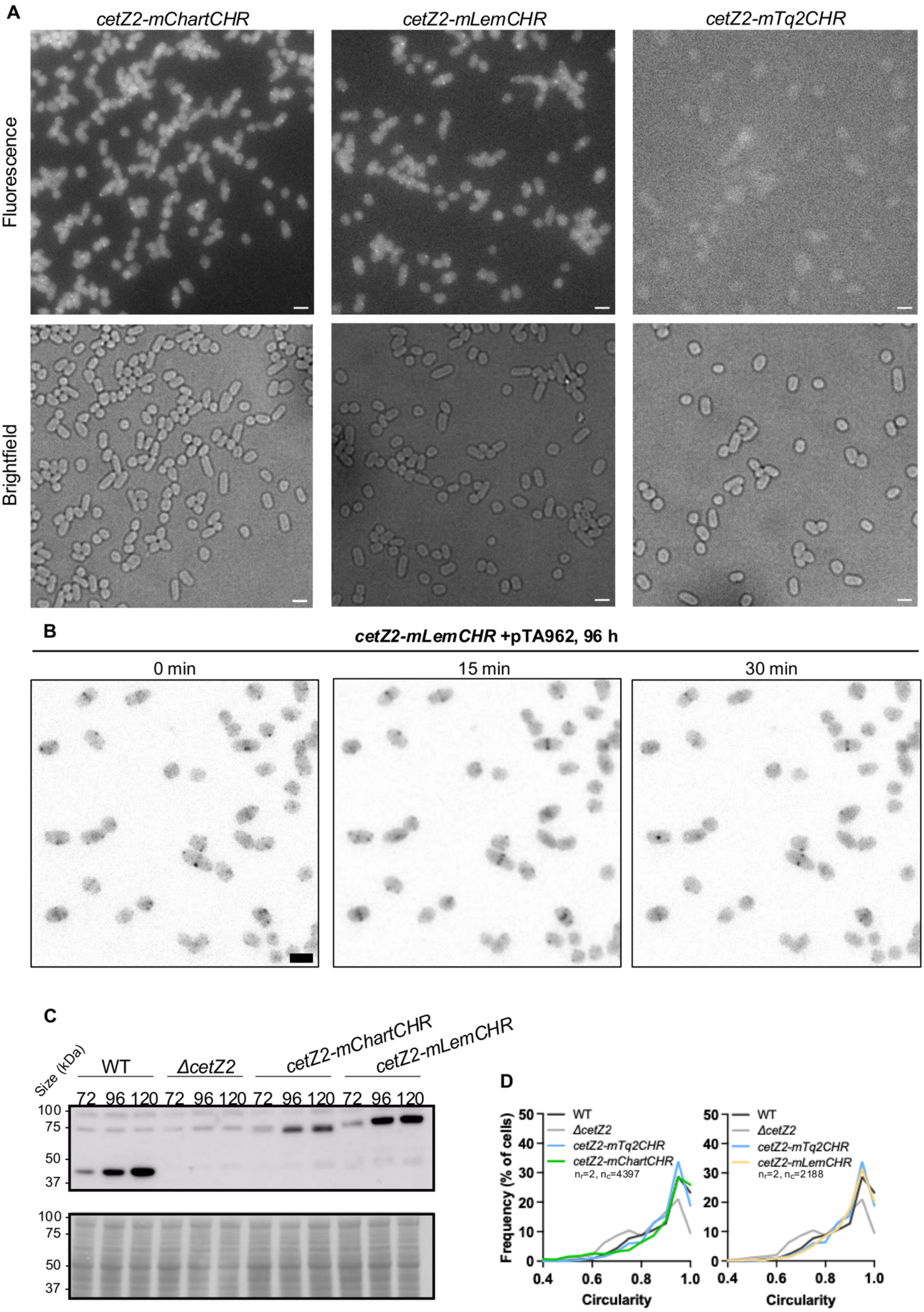
A
cetZ2-mChartCHR
cetZ2-mLemCHR
cetZ2-mTq2CHR
Fluorescence
Brightfield
B
cetZ2-mLemCHR +pTA962, 96 h
0 min
15 min
30 min
C
Size (kDa)
WT
ΔcetZ2
cetZ2-mChartCHR
cetZ2-mLemCHR
72 96 120 72 96 120 72 96 120 72 96 120
100
75
50
37
D
WT
ΔcetZ2
cetZ2-mTq2CHR
cetZ2-mChartCHR
nr=2, nc=4397
cetZ2-mLemCHR
nr=2, nc=2188
Frequency (% of cells)
Circularity

Figure S5. ***Chromosomal tagging of CetZ2 with mChartreuse and mLemon.*** **A)** *cetZ2-mChartCHR* +pTA962, *cetZ2-mLemCHR* + pTA962, and *cetZ2-mTq2CHR* + pTA962 were grown in Hv-YPCab medium and imaged at 96 h using brightfield and fluorescence microscopy. Brightness and contrast settings are maintained between panels. **B)** During mid-stationary phase (96 h), *cetZ2-mLemCHR* + pTA962 was imaged for 30 minutes in 15-minute intervals. In **A** and **B** scale bar=2 µm. **C)** Western blotting analysis using an antibody against CetZ2 on whole-cell lysate samples of *cetZ2-mChartCHR* + pTA962 and *cetZ2-mLemCHR* +pTA962 compared to wild-type + pTA962 and Δ*cetZ2* +pTA962 to assess proteolytic cleavage and upregulation of mChartreuse and mLemon fusions in stationary phase. **D)** Circularity of *cetZ2-mChartCHR* + pTA962 (left) and *cetZ2-mLemCHR* +pTA962 (right) during mid-stationary phase (96 h) was measured using phase-contrast microscopy of cultures shown in **A** (not shown) and cell shape analysis as described in the methods. Circularity distributions of wild-type + pTA962, Δ*cetZ2* +pTA962, and *cetZ2-mTq2CHR* + pTA962 from Figure S2a are included in both panels for comparison. The number of independent replicates analysed (n_r) and number of cells pooled from those replicates for analysis (n_c) are shown in the key.

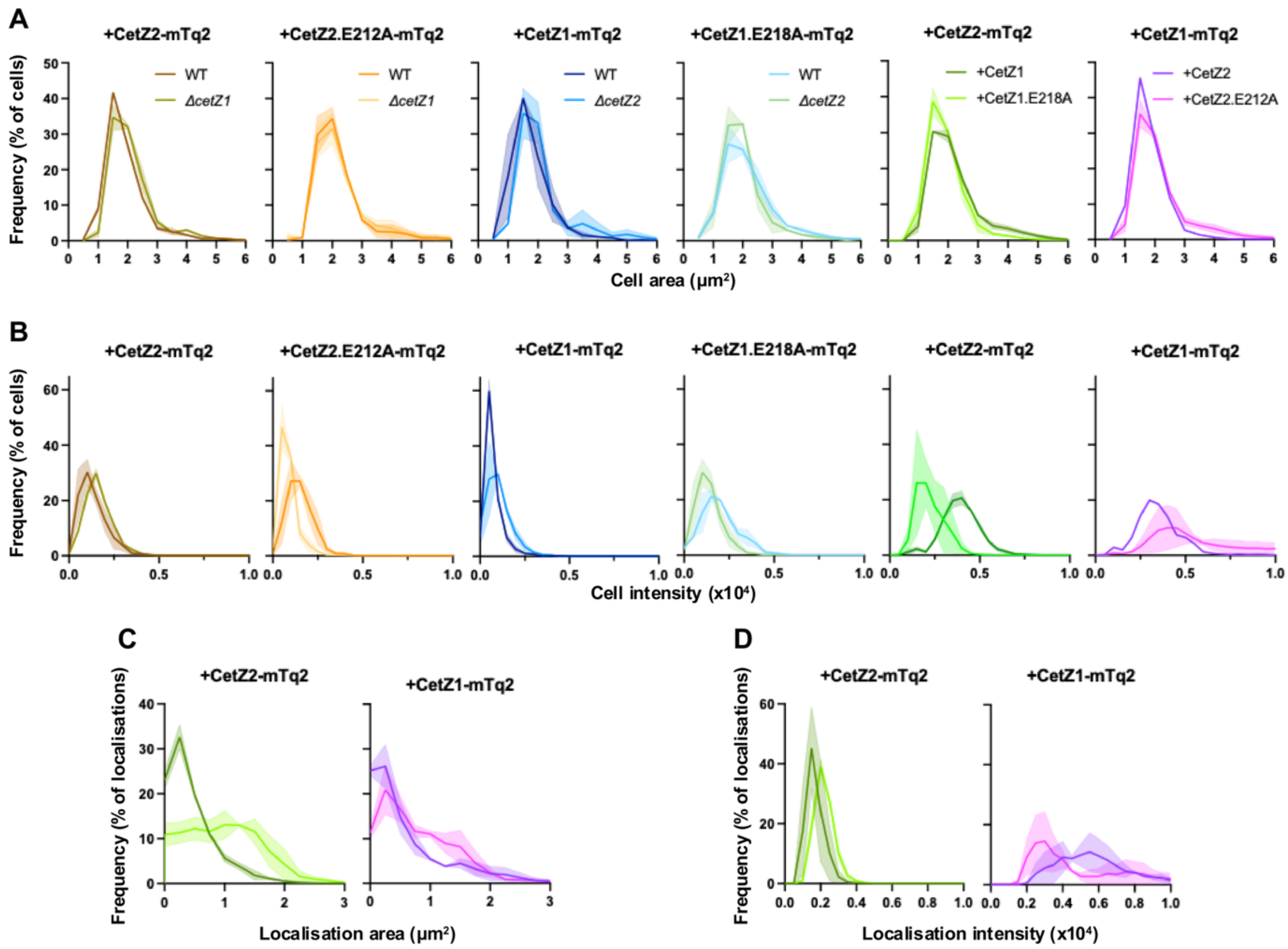

Figure S6. Extended analysis of CetZ1, CetZ2, CetZ1.E218A, and CetZ2.E212A localisation during mid-stationary phase. Datasets used for analysis in this figure are identical to those used in Figures 5 and 6, where the number of culture replicates (n_r), number of individual cells (n_c) and number of localisations (n_L) analysed are denoted. The key displayed in **A** applies to all panels. Frequency distributions of **A)** cell area, **B)** mean fluorescence intensity within the cell area (sum of pixel intensities normalised by the number of pixels within the cell boundary), **C)** localisation area, and **D)** mean localisation intensity (sum of pixel intensities normalised by the number of pixels within the boundary of a detected localisation).

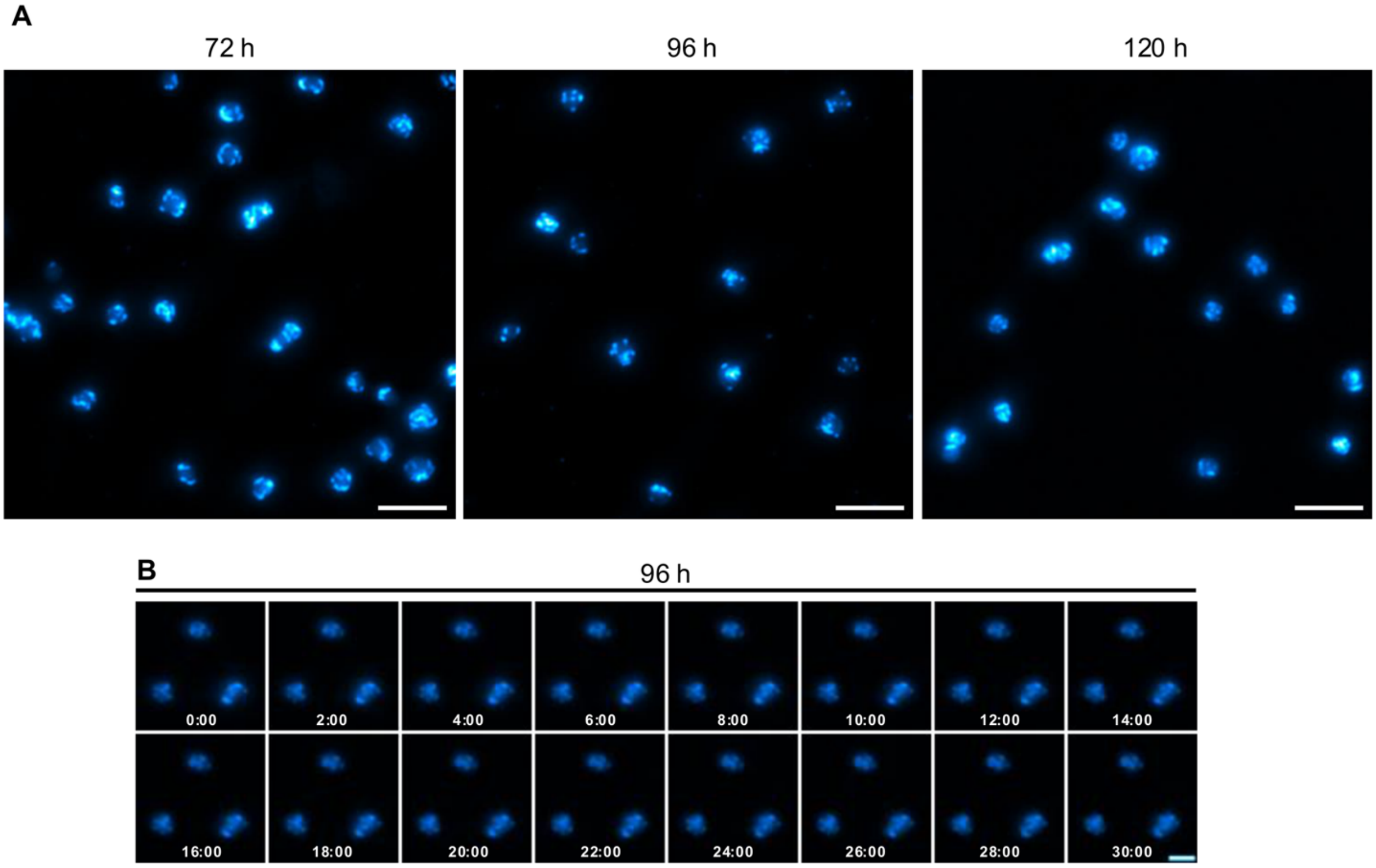

Figure S7. ***A mutation in the CetZ2 GTPase active site alters subcellular localization and blocks dynamic movement in stationary phase.*** **A)** WT + pHJB12 (CetZ2.E212A-mTq2) was grown in Hv-YPCab medium supplemented with 0.2 mM ʟ-tryptophan and imaged at 72, 96, and 120 h. Scale bar=5 µm. **B)** Time-lapse imaging of cells after 96 h. Images were taken at 1 min intervals for 30 min (SV. 5). Scale bar=2 µm. Images are brightness and contrast adjusted for viewability.

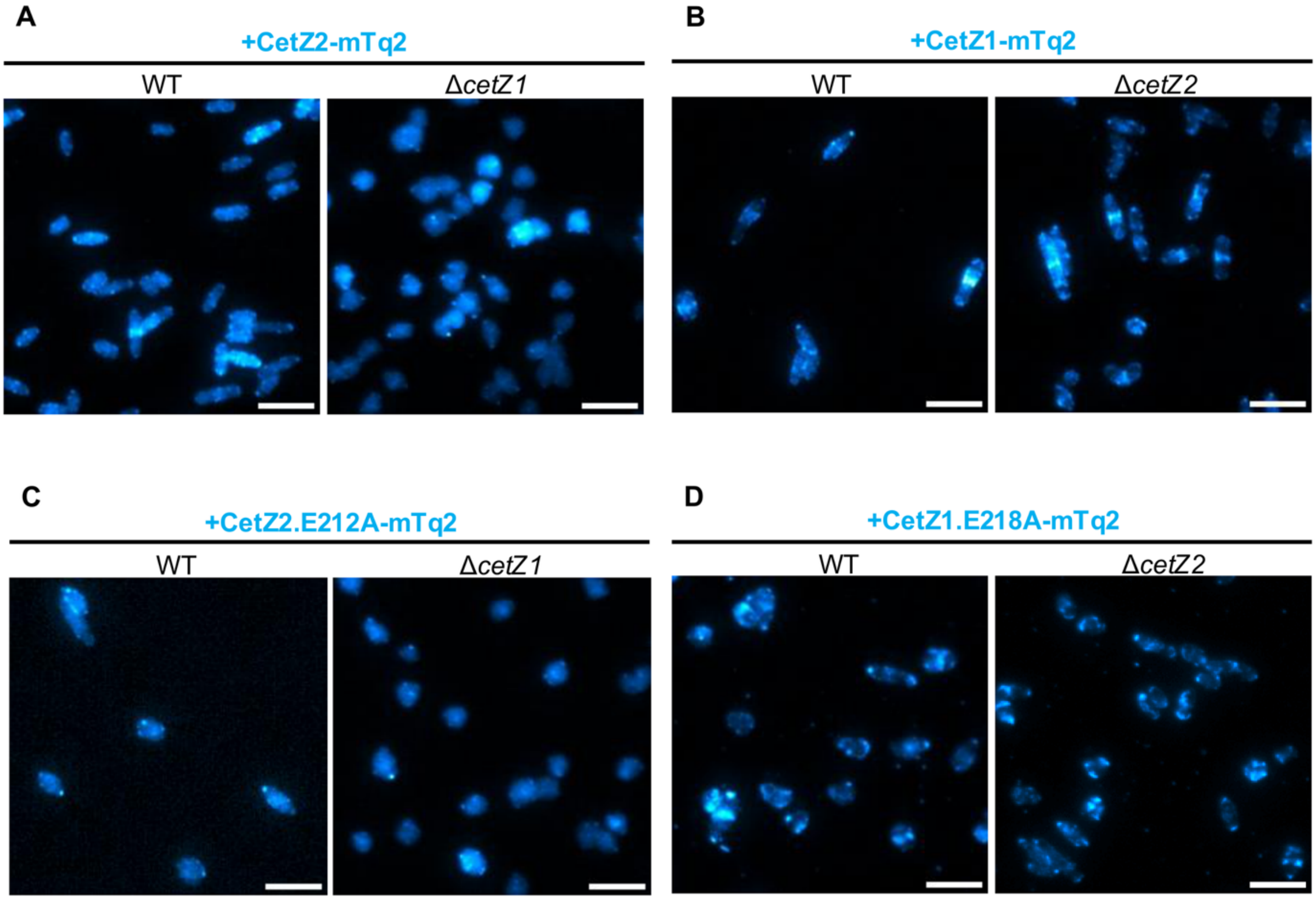

Figure S8. ***Localisation of CetZ1, CetZ2, CetZ1.E218A, and CetZ2.E212A during mid-log growth.*** All strains were grown in Hv-YPCab medium supplemented with 0.2 mM ʟ-tryptophan and imaged at 24 h. Scale bar=5 μm. **A)** WT and Δ*cetZ1* + pHJB6 (CetZ2-mTq2), **B)** WT and Δ*cetZ2* + pHVID135 (CetZ1-mTq2), **C)** WT and Δ*cetZ1* + pHJB12 (CetZ2.E212A-mTq2), and **D)** WT and Δ*cetZ2* + pHJB13 (CetZ1.E218A-mTq2). Images are brightness and contrast adjusted.

Table S1. Strains used in this study

**Strain**	**Genotype**	**Source**
ID621 H26	Δ*pyrE2,* cured of pHV2	[1]
ID622 Δ*cetZ1*	Δ*cetZ1* Δ*pyrE2,* cured of pHV2	[2]
ID357 Δ*cetZ2*	Δ*cetZ2* Δ*pyrE2,* cured of pHV2	[2]
ID643 *cetZ2-mTq2CHR*	*cetZ2-EG-mTq2* Δ*pyrE2,* cured of pHV2	This study
ID952 *cetZ2-mChartCHR*	*cetZ2-EG-mChartreuse* Δ*pyrE2,* cured of pHV2	This study
ID954 *cetZ2-mLemCHR*	*cetZ2-EG-mLemon* Δ*pyrE2,* cured of pHV2	This study

Table S2. Plasmids used in this study

**Plasmid**	**Description**	**Source**
pTA131_2204_IF	For in frame deletion of *cetZ1*	[3]
pTA131-*cetZ2*	For in frame deletion of *cetZ2*	[4]
pTA131-*cetZ2mTq2*	For chromosomal insertion of EG linker and mTurquoise2 at the C-terminus of *cetZ2*	This study (Genscript)
pTA131-*cetZ2mChart*	For chromosomal insertion of EG linker and mChartreuse at the C-terminus of *cetZ2*	This study (Genscript)
pTA131-*cetZ2mLem*	For chromosomal insertion of EG linker and mLemon at the C-terminus of *cetZ2*	This study (Genscript)
pTA962	Expression vector containing the p.*tna* promotor, *pyrE2* and *hdrB* genetic markers.	[5]
pTA962-*cetZ1*	For expression of *cetZ1* under the control of the p.*tna* promotor	[4]
pTA962-*cetZ1*.E218A	For expression of *cetZ1*.E218A under the control of the p.*tna* promotor	[4]
pTA962-*cetZ2*	For expression of *cetZ2* under the control of the p.*tna* promotor	[4]
pTA962-*cetZ2*.E212A	For expression of *cetZ2*.E212A under the control of the p.*tna* promotor	[4]
pHVID20	pTA962 based plasmid containing EG linker and YPet ORF, used to make pHJB5 and pHJB11	[3]
pHVID21	pTA962 based plasmid containing EG linker and mTurquoise2 ORF, used to make pHJB6 and pHJB12	[3]
pHVID135	For expression of *cetZ1*-mTq2 (containing G-linker region) under the control of the p.*tna* promotor	[3]
pHJB13	For expression of *cetZ1*.E218A-mTq2 (containing G-linker region) under the control of the p.*tna* promotor	[6]
pHJB6	For expression of *cetZ2*-mTq2 (containing EG-linker region) under the control of the p.*tna* promotor	[2]
pHJB12	For expression of *cetZ2*.E212A-mTq2 (containing EG-linker region) under the control of the p.*tna* promotor	This study
pHJB5	For expression of *cetZ2*-YPet (containing EG-linker region) under the control of the p.*tna* promotor	This study
pHJB11	For expression of *cetZ2*-YPet (containing EG-linker region) under the control of the p.*tna* promotor	This study
pHJB16	For dual expression of *cetZ2*-YPet (containing EG-linker region) and *cetZ1*-mTq2 (containing G-linker region) under the control of the p.*tna* promotor	This study
pHJB14	For dual expression of *cetZ2*-YPet (containing EG-linker region) and *cetZ1*.E218A-mTq2 (containing G-linker region) under the control of the p.*tna* promotor	This study
pHJB18	For dual expression of *cetZ2*.E212A-YPet (containing EG-linker region) and *cetZ1*-mTq2 (containing G-linker region) under the control of the p.*tna* promotor	This study
pHJB43	For dual expression of untagged *cetZ2* and *cetZ1*-mTq2 (containing G-linker region) under the control of the p.*tna* promotor	This study
pHJB44	For dual expression of untagged *cetZ2*.E212A and *cetZ1*-mTq2 (containing G-linker region) under the control of the p.*tna* promotor	This study
pHJB45	For dual expression of *cetZ2*-mTq2 (containing EG-linker region) and untagged *cetZ1* under the control of the p.*tna* promotor	This study
pHJB46	For dual expression of *cetZ2*-mTq2 (containing EG-linker region) and untagged *cetZ1*.E218A under the control of the p.*tna* promotor	This study

**Table S3.** Oligonucleotides used in this study		
**Name**	**Sequence 5’-3’**	**Description/use**
HfxZ3_F	CCCCCGGGAATTCATATGAAAACCGTCCTGATTG GTGTGGGG	Forward primer for amplification of *cetZ2*/*cetZ2*.*E212A* ORFs
HfxZ3_RnoS	CGCGGATCCTCACAGCAGGTCGTCGAGGTCGTC	Reverse primer for amplification of *cetZ2*/*cetZ2*.*E212A* ORFs, excluding the stop codon
BglII_CetZ1	GGCGGCGCTAGCATGAAGCTCGCAATGATCG	Forward primer for amplification of *cetZ1*/*cetZ1*.*E212A* ORFs, incorporating BglII at the 5’ end
NheI_CetZ1	GGCGGGCGCTAGCATGAAGCTCGCAATGATC	Forward primer for amplification of *cetZ1*/*cetZ1*.*E212A* ORFs, incorporating NheI at the 5’ end
T3	GCAATTAACCCTCACTAAAGG	Reverse primer for amplification of *cetZ1*/*cetZ1*.*E218A* ORFs.

Legends for Supplementary Videos:

Video 1. **3D render of CetZ2-mTq2 in an *H. volcanii* cell.** CetZ2-mTq2 was produced from pHJB6 in the wild-type background and cells were sampled from mid-stationary phase culture after 96 h of culturing in Hv-YPCab medium supplemented with 0.2 mM L-tryptophan. The image was acquired with 3D-SIM and 3D rendered in Imaris. Scale bar=0.5 µm.

Video 2. **Time-lapse imaging of CetZ2-mTq2 localization dynamics in early, mid and late stationary phase cells.** CetZ2-mTq2 was produced in the wild-type background from pHJB6. Cells were grown in Hv-YPCab medium supplemented with 0.2 mM L-tryptophan and sampled in early, mid-, and late stationary phase (72, 96, and 120 h, respectively) for time-lapse microscopy. Images were taken at 1 min intervals for 30 min. Scale bar=5 µm.

Video 3. **Tracking of CetZ2-mTq2 localizations in *H. volcanii* stationary phase cells.** Examples of localization tracking using TrackMate2 to analyse dynamics of CetZ2-mTq2. CetZ2-mTq2 was produced from pHJB6 in the wild-type background, during mid-stationary phase (96 h). Individual cells are shown without (left) and with (right) traces of the tracked trajectories (shown in yellow) generated by TrackMate2. Scale bars=5 µm.

Video 4. **Time-lapse imaging of tagged CetZ2 produced from its native chromosomal locus in stationary phase cells.** *H. volcanii cetZ2-mChartCHR* and *cetZ2-mLemCHR* producing CetZ2-mChartreuse (left) and CetZ2-mLemon (right) were grown in Hv-YPCab medium and sampled in mid-stationary phase (96 h). Fluorescence images were taken at 15 min intervals for 30 min. Scale bar=5 µm.

Video 5. **Time-lapse imaging of CetZ2-mTq2 and CetZ2-E212A-mTq2 in the presence or absence of CetZ1 in stationary phase cells.** Time-lapse microscopy showing CetZ2-mTq2 produced in the wildtype background (top-left) and Δ*cetZ1* background (top-right) from pHJB6. Time-lapse microscopy showing CetZ2.E212A-mTq2 produced in the wildtype background (bottom-left) and Δ*cetZ1* background (bottom-right) from pHJB12. Cells were grown in Hv-YPCab medium supplemented with 0.2 mM L-tryptophan and sampled in mid-stationary phase (96 h). Images were taken at 1 min intervals for 30 min. Scale bar=5 µm.

Video 6. **Time-lapse imaging of the GTPase mutant CetZ2.E212A-mTq2 in stationary phase cells.** *H. volcanii* producing CetZ2.E212A-mTq2 (from pHJB12) in the wildtype background were grown in Hv-YPCab medium supplemented with 0.2 mM L-tryptophan and sampled in mid-stationary phase (96 h) for time-lapse fluorescence microscopy. Images were taken at 1 min intervals for 30 min. Scale bar=5 µm.

Video 7. **Time-lapse imaging of CetZ1-mTq2 and CetZ1-E218A-mTq2 in stationary phase cells in the presence or absence of CetZ2.** CetZ1-mTq2 was produced in the wildtype background (top-left) and Δ*cetZ2* background (top-right) from pHVID135.

CetZ1.E218A-mTq2 was produced in the wildtype background (bottom-left) and Δ*cetZ2* background (bottom-right) from pHJB13.Cells were grown in Hv-YPCab medium supplemented with 0.2 mM L-tryptophan and sampled in mid-stationary phase (96 h). Images were taken at 1 min intervals for 30 min. Scale bar=5 μm.

Video 8. **Time-lapse imaging of CetZ1-mTq2 or CetZ2-mTq2 in stationary phase cells co-producing the alternate CetZ or GTPase mutant.** Cells of the wild-type background containing pHJB43 to produce CetZ1-mTq2 and CetZ2 (top-left), pHJB44 to produce CetZ1-mTq2 and CetZ2.E212A (top-right), pHJB45 to produce CetZ2-mTq2 and CetZ1 (bottom-left) or pHJB46 to produce CetZ2-mTq2 and CetZ1.E218A (bottom-right) were grown in Hv-YPCab medium supplemented with 0.2 mM L-tryptophan and sampled in mid-stationary phase (96 h). Images were then taken at 1 min intervals for 30 min. Scale bar=5 μm.

References:

1. Allers T, Ngo H-P, Mevarech M, Lloyd RG: **Development of additional selectable markers for the halophilic archaeon Haloferax volcanii based on the leuB and trpA genes**. *Applied and environmental microbiology* 2004, **70**(2):943-953.
2. Brown HJ, Islam MI, Ruan J, Baker MA, Ithurbide S, Duggin IG: **CetZ1-dependent assembly and positioning of the motility machinery in haloarchaea**. *bioRxiv* 2024:2024.2005. 2002.592137.
3. Ithurbide S, de Silva RT, Brown HJ, Shinde V, Duggin IG: **A vector system for single and tandem expression of cloned genes and multi-colour fluorescent tagging in Haloferax volcanii**. *Microbiology* 2024, **170**(5).
4. Duggin IG, Aylett CH, Walsh JC, Michie KA, Wang Q, Turnbull L, Dawson EM, Harry EJ, Whitchurch CB, Amos LA: **CetZ tubulin-like proteins control archaeal cell shape**. *Nature* 2015, **519**(7543):362.
5. Allers T, Barak S, Liddell S, Wardell K, Mevarech M: **Improved strains and plasmid vectors for conditional overexpression of His-tagged proteins in Haloferax volcanii**. *Applied and environmental microbiology* 2010, **76**(6):1759-1769.
6. de Silva RT, Shinde V, Brown HJ, Liao Y, Duggin IG: **Dynamic self-association of archaeal tubulin-like protein CetZ1 drives Haloferax volcanii morphogenesis**. *bioRxiv* 2024:2024.2004. 2008.588506.
